# A randomised crossover trial comparing photobiomodulation therapy with other recovery strategies in CrossFit athletes

**DOI:** 10.1371/journal.pone.0349880

**Published:** 2026-05-22

**Authors:** Paulo Henrique Gusmão Nogueira Martins, Shaiane Silva Tomazoni, Caroline dos Santos Monteiro Machado, Amanda Lima Pereira, Henrique Dantas Pinto, Matheus Marinho Aguiar Lino, Neide Firmo Ribeiro, Luana Barbosa Dias, Ivo de Oliveira Aleixo Junior, Marcelo Ferreira Duarte de Oliveira, Older Manoel de Araújo-Silva, Thiago De Marchi, Heliodora Leão Casalechi, Ernesto Cesar Pinto Leal-Junior

**Affiliations:** 1 Laboratory of Phototherapy and Innovative Technologies in Health (LaPIT), Post-graduate Program in Rehabilitation Sciences, Nove de Julho University, São Paulo, Brazil; 2 ELJ Consultancy, Scientific Consultants, São Paulo, Brazil; 3 Core Sport Fisioterapia, Caraguatatuba, Brazil; Complutense University of Madrid: Universidad Complutense de Madrid, SPAIN

## Abstract

**Background:**

This study compared the effects of different therapeutic modalities on recovery following the workout of the day (WOD) in CrossFit athletes, focusing on reducing muscle damage and improving functional performance.

**Methods:**

In a randomised, controlled, crossover design, twelve CrossFit athletes underwent four post-WOD recovery interventions across four weeks: passive recovery (PR), intermittent pneumatic compression (IPC), extracorporeal shock wave therapy (ESWT), and photobiomodulation therapy combined with a static magnetic field (PBMT-sMF). Assessments were conducted at baseline, and at 1, 24, and 48 hours post-WOD. The primary outcome was vertical jump performance (countermovement jump [CMJ] height) at 1-hour post-WOD; secondary outcomes included lactate dehydrogenase (LDH) levels, superoxide dismutase (SOD) and catalase (CAT) activity, thiobarbituric acid reactive substances (TBARS), carbonylated proteins, perceived exertion, and intervention satisfaction.

**Results:**

There was no significant difference between interventions in CMJ height at 1-hour post-WOD. In contrast, PBMT-sMF outperformed the other interventions in the secondary outcomes. PBMT-sMF significantly improved CMJ height at 24 and 48 hours compared with PR (p < 0.05) and led to greater reductions in LDH at both time points (p < 0.05 and p < 0.001, respectively). PBMT-sMF also produced lower TBARS and carbonylated protein levels, with higher CAT and SOD activities at 24 and 48 hours (p < 0.05 and p < 0.0001, respectively). CAT and SOD activities were also significantly elevated at 1 hour post-WOD compared with PR (p < 0.05).

**Conclusions:**

PBMT-sMF may be an effective recovery strategy for CrossFit athletes, with its benefits possibly related to the modulation of oxidative stress as an underlying mechanism.

**Trial registration:**

Trial registration number: NCT06628609.

## Introduction

Intense and/or prolonged physical exercise induces repeated eccentric contractions and tissue vibration [1,2]. These factors can lead to muscle damage and subsequent tissue inflammation, marked by elevated biochemical markers such as lactate dehydrogenase (LDH), creatine kinase (CK), interleukin-6 (IL-6), and C-reactive protein (CRP), along with delayed-onset muscle soreness (DOMS) and increased perceived fatigue [3–5]. High-intensity exercises, such as the workout of the day (WOD) in CrossFit training, can result in extreme fatigue [6].

Muscle fatigue refers to the reduction in force or power due to sustained contractions [7]. It results from dysfunction in neural, vascular, ionic, and metabolic systems, with fatigue-inducing agents such as hydrogen ions (H⁺), lactate, inorganic phosphate (Pi), heat shock proteins (HSP), and reactive oxygen species (ROS) contributing to performance decline [8,9]. At rest, skeletal muscle maintains low ROS levels, but intense exercise increases ROS production, leading to oxidative damage to nucleic acids, lipids, and proteins, alongside reduced antioxidant capacity [9–11]. Elevated oxidative damage impairs muscle function and prolongs recovery [12].

Photobiomodulation therapy (PBMT), particularly when combined with a static magnetic field (PBMT-sMF), has emerged as a promising approach to mitigate physiological stress induced by exercise [5]. PBMT is a non-ionising, non-thermal modality that uses lasers or light-emitting diodes (LEDs) in the visible to infrared spectrum [13]. Evidence indicates that PBMT-sMF can accelerate post-exercise recovery and enhance physical performance [5]. These ergogenic effects are thought to be mediated, at least in part, through modulation of oxidative stress, specifically by increasing superoxide dismutase (SOD) and catalase (CAT) activity [4,14–16], and reducing thiobarbituric acid reactive substances (TBARS) and carbonylated proteins [14–18]. Other modalities, such as intermittent pneumatic compression (IPC) and extracorporeal shock wave therapy (ESWT), are also used in recovery. IPC applies external pressure via inflatable garments (0–300 mmHg), enhancing lymphatic flow and clearing metabolic by-products associated with muscle damage and neuromuscular fatigue [19,20]. ESWT involves high-energy acoustic waves and promotes tissue healing, regeneration, and pain relief [21–23], with limited evidence for exercise recovery [24].

In recent years, the use of IPC and ESWT for post-exercise recovery and performance enhancement has gained popularity in the sports setting. However, despite this growing interest, evidence supporting their effectiveness in promoting recovery and improving performance remains scarce. In addition, their impact on oxidative stress resulting from high-intensity exercise has not been elucidated. Well-designed studies are essential to guide clinicians in selecting the most appropriate therapeutic modality for athletes, optimising effectiveness while avoiding unnecessary costs. In this context, it is particularly relevant to investigate whether these interventions can reduce muscle damage and fatigue, thereby improving functional performance. To our knowledge, no prior studies have compared PBMT-sMF, IPC, ESWT, and passive recovery in CrossFit athletes with these aims. Therefore, this study aimed to compare the effects of different therapeutic modalities on recovery following the WOD in CrossFit athletes, focusing on reducing muscle damage and improving functional performance.

## Materials and methods

### Study design and ethics

This randomised, controlled, single-blinded (outcome assessors), crossover trial was prospectively registered at ClinicalTrials.gov (NCT06628609). The study was approved by the Nove de Julho University Ethics Committee (Protocol 3.997.120) and conducted in accordance with Declaration of Helsinki. Written informed consent was obtained, and no protocol changes occurred.

### Participants

The trial was conducted in São José dos Campos, Brazil. The recruitment period extended from October 11, 2024, to November 3, 2024. It enrolled 12 healthy male CrossFit athletes (18–35 years), all non-professionals, who had been training at least four times/week for a minimum of one year. Exclusion criteria included pharmacological use or recent musculoskeletal injuries. Additionally, individuals who sustained musculoskeletal or joint injuries during the study were also excluded.

### Interventions

All interventions were applied five minutes post-WOD and lasted 30 minutes, except for PBMT-sMF, which was applied for 32 minutes.

- Passive Recovery (PR): Participants remained at rest in the supine position throughout the intervention period, serving as the control condition [25].
- Intermittent Pneumatic Compression (IPC): Sequential compression was delivered using pneumatic boots (model 701 RA, Recovery Pump LLC, Concordville, PA, USA), positioned on both lower limbs. The device applied a pressure of 80–90 mmHg in four inflatable chambers arranged linearly from the feet to the hips. Each chamber was sequentially inflated for approximately 8–12 seconds, followed by a partial deflation period of 15 seconds, with the full inflation cycle lasting between 30 and 40 seconds [26].
- Extracorporeal Shock Wave Therapy (ESWT): The intervention was administered using the Shock Wave Therapy BTL-6000 device (BTL Industries Ltd., United Kingdom), following the manufacturer’s instructions. Each session involved the delivery of 2,000 pulses per region (6,000 pulses per lower limb), with an energy flux density of 0.03 mJ/mm², a frequency of 10 Hz, and a pressure of 1.5 bar [24]. A 15 mm diameter acoustic wave transmitter was applied directly to the skin using a conductive gel to optimise (*optimize*) coupling and energy distribution [24]. ESWT was targeted at the knee extensor/hip flexor muscles (rectus femoris, vastus lateralis, and vastus medialis), knee flexor/hip extensor muscles (hamstrings), and plantar flexor muscles (gastrocnemius) in both lower limbs. The application sites and treatment protocol were based on a previous study and manufacturer guidelines to ensure optimal effectiveness [24].
- Photobiomodulation Therapy Combined with a Static Magnetic Field (PBMT-sMF): This intervention was performed using a Multi Radiance Medical device (Solon, OH, USA) equipped with a cluster probe that emits a negligible amount of heat [27]. The device contained 20 diodes, distributed as follows: four diodes emitting at 905 nm (1.25 mW average power, 50 W peak power per diode), eight diodes emitting at 850 nm (40 mW average power per diode), and eight diodes emitting at 633 nm (25 mW average power per diode). The PBMT-sMF was applied to eight specific sites in both lower limbs, targeting: Four sites in the knee extensor/hip flexor muscles (rectus femoris, vastus lateralis, and vastus medialis); Three sites in the knee flexor/hip extensor muscles (hamstrings); One site in the plantar flexor muscles (gastrocnemius). The parameters used for PBMT-sMF were selected based on prior research to ensure consistency and therapeutic effectiveness [5,16,28]. A comprehensive list of PBMT-sMF parameters is provided in Table 1.

**Table 1:** PBMT-sMF parameters.

	Lasers	Red LEDs	Infrared LEDs
Number of diodes	4	8	8
Wavelength (nm)	905	633	850
Frequency (Hz)	250	2	250
Peak power (W)	50	–	–
Average optical output (mW)- each	1.25	25	40
Power density (mW/cm^2^) – each	3.91	29.41	71.23
Energy density (J/cm^2^) – each	0.50*; 0.44**; 0.44***	3.79*; 3.39**; 3.39***	9.21*; 8.21**; 8.21***
Dose (J) – each	0.16*; 0.14**; 0.14***	3.22*; 2.88**; 2.88***	5.16*; 4.60**; 4.60***
Spot size of diode (cm^2^) – each	0.32	0.85	0.56
Magnetic field (mT)	110		
Irradiation time per site (sec)	129*; 115**; 115***		
Total dose per site (J)	67.68*; 60.76**; 60.76***		
Total dose applied per lower limb (J)	270.72*; 182.28**; 60.76***		
Aperture of device	33		
Application mode	Direct skin contacts and slight pressure		

* Knee extensors, ** knee flexors, *** plantar flexors.

### Blinding and randomization

Outcomes were assessed by an independent evaluator blinded to group allocation. Intervention sequences were randomised (1:1:1:1) using www.randomization.org by a researcher not involved in other study phases. In the first phase, participants were allocated so that each intervention received 25% of the sample. In the subsequent phases, the same procedure was applied to ensure that each of the four interventions included one quarter of the participants, thereby maintaining an equal distribution across all phases of the trial. Each participant completed all four interventions across weekly sessions with seven-day washouts. Allocation was concealed using sealed, numbered opaque envelopes. The intervention orders were:

- Sequence 1: PR → PBMT-sMF → ESWT → IPC
- Sequence 2: PBMT-sMF → ESWT → IPC → PR
- Sequence 3: ESWT → IPC → PR → PBMT-sMF
- Sequence 4: IPC → PR → PBMT-sMF → ESWT

### Study sessions and exercise protocol

This trial comprised four sessions with seven-day washouts. Procedures remained identical, except for the randomised intervention applied. Assessments occurred before and after each WOD.

- Exercise Protocol – WOD: To minimize circadian and external influences, participants performed the WOD at the same time each session and avoided physical activity from 24 h pre-WOD to 48 h post-WOD. The WOD followed the 21-15-9 CrossFit scheme (three rounds, no rest), aiming for the fastest completion. Repetitions were monitored by an independent assessor. Exercises included:
  1. Assault AirBike Calories: Participants used a standardized AirBike model, pedaling until they achieved the target calorie count required for each round.
  2. Hang Squat Clean: The exercise began with the participant holding the barbell in a hang position (above the knees) with fully extended arms. From this position, the barbell was cleaned to the shoulders, while the hips moved below the knees. A successful repetition was counted when the participant achieved full knee and hip extension, with the barbell resting on the shoulders.
  3. Box Jump Over: Participants performed a two-foot jump onto a 60.69 cm (24-inch) box, ensuring both feet made contact with the top surface before jumping down onto the opposite side.

### Outcomes

The primary outcome of this study was the change in performance on the functional test assessed 1 hour post-WOD. Secondary outcomes included the change in performance on the functional test at 24 and 48 hours post-WOD, as well as biochemical markers of muscle damage and oxidative stress, antioxidant activity, and subjective perception of exertion evaluated at 1, 24 and 48 hours post-WOD. Finally, satisfaction with the intervention was evaluated at 48 hours post-WOD.

- Vertical Jump Performance: Vertical jump performance was measured using the countermovement jump (CMJ) height test. [29] During the test, participants stood with their feet aligned at shoulder width and hands placed on their hips. They then performed a controlled squat until reaching approximately 90° of knee flexion, followed by an explosive vertical jump. Participants were instructed to flex the hips, knees, and ankles upon landing to absorb impact. A smartphone camera, positioned 1.5 metres away, recorded three maximal-effort CMJ attempts [29]. Jump height was analysed using the JumPo 2 mobile application (available for iOS devices), as it was the only validated tool accessible during data collection. The smartphone-based CMJ assessment method employed in this study has been previously tested for validity and reliability, demonstrating strong agreement with gold-standard force plate measurements [29]. The highest recorded jump was considered for analysis.
- Muscle Damage, Oxidative Stress Markers: Muscle damage was assessed via lactate dehydrogenase (LDH) levels [4], while oxidative stress and antioxidant activity were evaluated through thiobarbituric acid reactive substances (TBARS), carbonylated protein, catalase (CAT), and superoxide dismutase (SOD) activities [16]. Blood samples (5 mL) were drawn from the anterior cubital vein and stored at −80°C for subsequent analysis. LDH activity was quantified using spectrophotometry with specific reagent kits (Labtest®, Minas Gerais, Brazil) as an indirect marker of muscle damage. TBARS, carbonylated proteins, CAT, and SOD were analysed as biomarkers of oxidative stress and antioxidant defence, following previously established spectrophotometric protocols [16]. Each biochemical analysis was conducted in triplicate, and the mean value was used for statistical interpretation.
- Subjective Perception of Exertion: Perceived exertion was evaluated using the Rate of Perceived Exertion (RPE) scale [30,31]. Participants rated their exertion in two specific domains: RPE-MI (Muscular Intensity): Perceived fatigue in the lower limbs and RPE-R (Respiratory Effort): Perceived cardiorespiratory fatigue. Volunteers completed two identical RPE scales, each ranging from 0 to 100, corresponding to their muscular and cardiorespiratory effort levels.
- Satisfaction with the Intervention: Participant satisfaction with the intervention was measured 48 hours post-WOD using a 5-point Likert scale. Volunteers rated their satisfaction as follows: (1) Very unsatisfied, (2) Unsatisfied, (3) Neutral (neither satisfied nor unsatisfied), (4) Satisfied, and (5) Very satisfied. For analysis, responses were dichotomised into two categories: Satisfied (including “satisfied” and “very satisfied”) and not satisfied (including “neutral,” “unsatisfied,” and “very unsatisfied”). This approach was adopted to distinguish participants who expressed clear satisfaction from those who did not, as neutral responses are generally interpreted as the absence of a positive evaluation rather than satisfaction.

A detailed overview of all study procedures and assessments is provided in the Supporting Information (S1 Fig).

### Statistical analysis

To our knowledge, no prior studies have compared PBMT-sMF, IPC, ESWT, and passive recovery in CrossFit athletes. Thus, the sample size was estimated based on CMJ performance data obtained from the first session of the trial, following a previously described method [16]. Therefore, this represents a post hoc calculation rather than a true a priori sample size determination. Twelve participants (three per intervention sequence) were included. This calculation, conducted by a blinded researcher, was based on CMJ percentage changes at 1 hour post-WOD (the primary outcome), expressed as mean ± standard deviation: PBMT-sMF: 102.12% (±5.51); ESWT: 94.12% (±5.72); IPC: 95.16% (± 6.36); PR: 95.98% (± 5.04). The resulting effect size (1.31) indicated that 12 participants ensured 80% power (α = 0.05; ANOVA), as confirmed via WebPower (https://webpower.psychstat.org/models/means03/effectsize.php) and Statistics Kingdom tools (https://www.statskingdom.com/sample_size_regression.html). Given the crossover design, this sample was considered adequate.

Statistical analyses followed the intention-to-treat principle [32]. The statistician was blinded to allocation to minimise bias. Data normality was assessed using the Shapiro-Wilk test. Differences between interventions were evaluated using repeated-measures ANOVA with Bonferroni post hoc tests. The assumption of sphericity was assessed using Mauchly’s test. As no violations were detected, Greenhouse–Geisser corrections were not required; detailed results (including epsilon values and corrected degrees of freedom) are provided in the Supporting Information (S1 Table). Both absolute values and percentage changes from baseline were analysed. Results are presented as mean ± SD (tables) and mean ± SEM (figures). Fisher’s exact test compared satisfaction proportions across interventions. Significance was set at p < 0.05. All statistical analyses were performed using GraphPad Prism, version 10.6.1 (GraphPad Software, San Diego, CA, USA). Effect sizes for between-intervention pairwise comparisons were calculated as Cohen’s *d* for paired (dependent) samples, given the crossover design. Effect size magnitudes were interpreted according to Cohen’s conventional thresholds: 0.2 (small), 0.5 (moderate), and 0.8 (large). All calculations were performed using G*Power (version 3.1.9.6). Detailed between-group comparisons, including mean differences, 95% confidence intervals, p-values, and effect sizes, are provided in the Supporting Information (S2 Table). All data underlying the findings are available in the Supporting Information (S1 Dataset).

## Results

**Fig 1:**
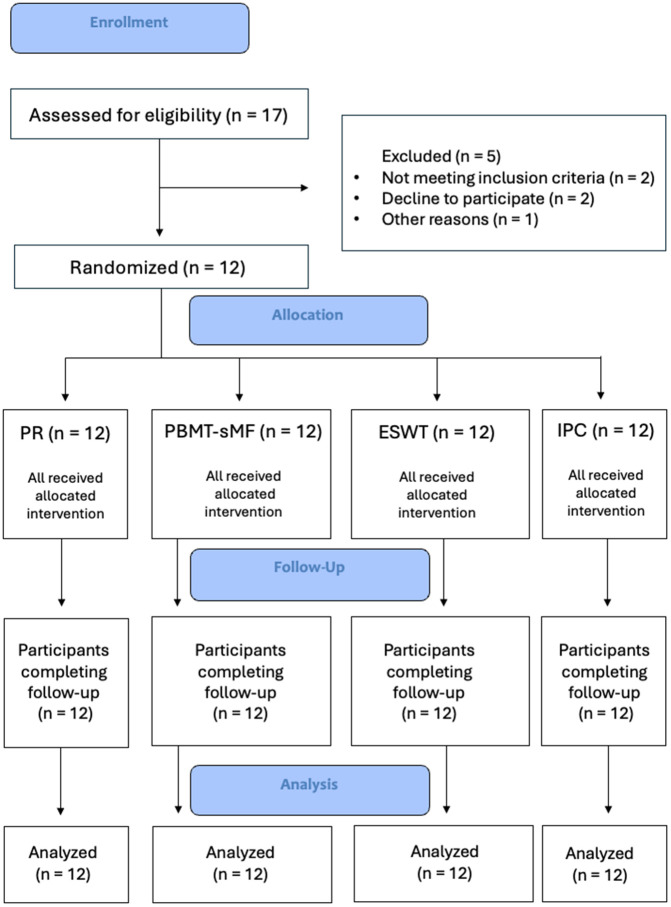
CONSORT flowchart of the study.

A total of twelve male CrossFit athletes (n = 12) successfully completed all study procedures (Fig 1). The study was conducted between October 2024 and November 2024. The participants had a mean age of 29.7 years (±3.2), a height of 177 cm (±7), a weight of 87 kg (±11), and a body mass index (BMI) of 27.8 kg/m² (±2.3). The mean duration of their CrossFit training experience was 37 months (±21). No adverse events were reported by any of the participants throughout the trial. Detailed statistical comparisons are provided in the Supporting Information (S2 Table).

### WOD duration and consistency across sessions

WOD completion times did not differ significantly across study weeks (p > 0.05), suggesting no learning effect. Weekly means were: Week 1–557.33 s (±116.86), Week 2–526.83 s (±91.19), Week 3–503.50 s (±66.48), Week 4–500.17 s (±94.58). Similarly, no significant differences were found between interventions (p > 0.05), confirming comparable conditions: PR – 512.25 s (±90.64), IPC – 509.42 s (±82.36), ESWT – 547.17 s (±128.26), PBMT-sMF – 519.00 s (±72.05).

### Functional performance

**Fig 2:**
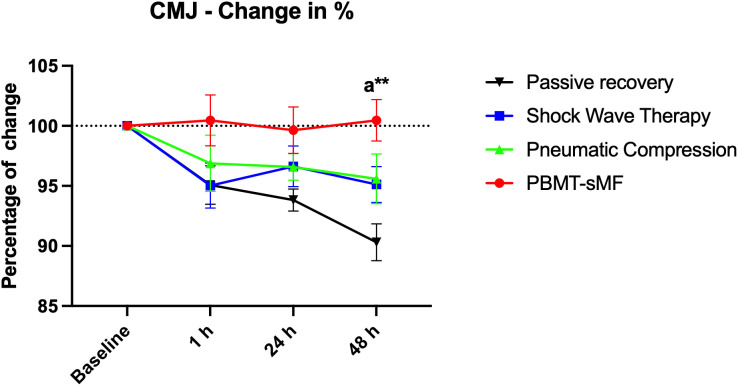
Percentage change in CMJ performance. The data are presented as mean and SEM. a: vs passive recovery; *p < 0.05; ***p < 0.001. Statistical test: mixed design ANOVA with Bonferroni post hoc test.

There was no significant difference between interventions in vertical jump performance (CMJ height) at 1-hour post-WOD (primary outcome). However, a statistically significant improvement in vertical jump performance (CMJ height) was observed following the PBMT-sMF intervention compared with PR. Specifically, CMJ height was higher after PBMT-sMF at 24 hours post-WOD (p = 0.0431) and at 48 hours post-WOD (p < 0.0001) (Fig 2).

### Muscle damage

**Fig 3:**
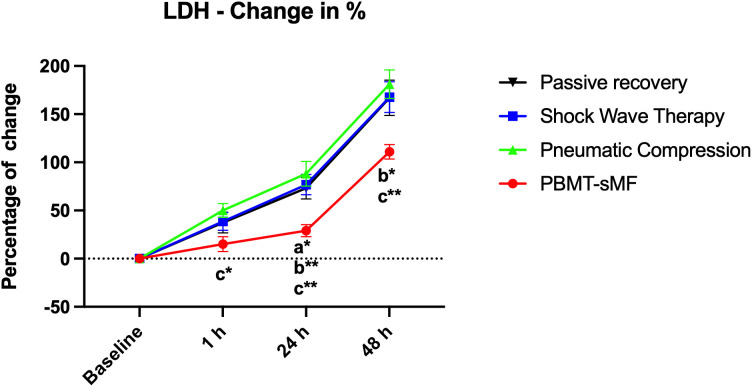
Percentage change in LDH. The data are presented as mean and SEM. a: vs passive recovery; b: vs ESWT; c: vs IPC; *p < 0.05; **p < 0.01; ***p < 0.001; ****p < 0.0001. Statistical test: mixed design ANOVA with Bonferroni post hoc test.

LDH levels were significantly lower following PBMT-sMF compared with PR (p = 0.0131), ESWT (p = 0.0057), and IPC (p = 0.0003) at 24 hours post-WOD. This advantage persisted at 48 hours, with PBMT-sMF showing lower LDH levels than PR (p = 0.0007), ESWT (p = 0.0006), and IPC (p < 0.0001) (Fig 3).

### Oxidative stress markers

**Fig 4:**
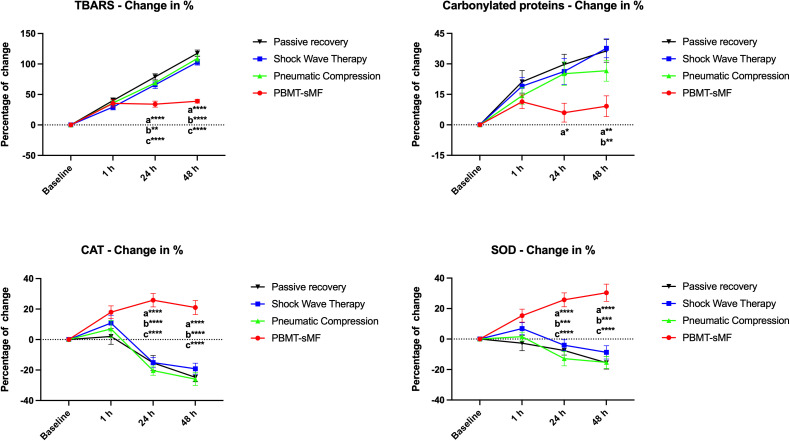
Percentage change in TBARS, carbonylated proteins, CAT, and SOD. The data are presented as mean and SEM. a: vs passive recovery; b: vs ESWT; c: vs IPC; *p < 0.05; **p < 0.01; ***p < 0.001; ****p < 0.0001. Statistical test: mixed design ANOVA with Bonferroni post hoc test.

TBARS levels were significantly lower following PBMT-sMF compared with PR, ESWT, and IPC at both 24 and 48 hours post-WOD (p < 0.0001 for all comparisons) (Fig 4). For carbonylated proteins, PBMT-sMF showed significantly lower levels than PR (p = 0.001), ESWT (p = 0.0072), and IPC (p = 0.0127) at 24 hours. This difference persisted at 48 hours, with PBMT-sMF presenting lower values than PR (p = 0.0001), ESWT (p < 0.0001), and IPC (p = 0.0307) (Fig 4). For antioxidant activity, CAT and SOD levels were significantly higher at 1-hour post-WOD after PBMT-sMF compared with PR (p = 0.0187 and p = 0.0038, respectively). These differences remained at 24 and 48 hours, with PBMT-sMF maintaining higher CAT and SOD activity than PR, ESWT, and IPC (p < 0.0001 for all comparisons) (Fig 4).

### Participants’ satisfaction with the intervention

Satisfaction rates were 42% for PR, 83% for IPC, 83% for ESWT, and 67% for PBMT-sMF. Satisfaction was significantly higher for IPC (p = 0.0195) and ESWT (p = 0.0125) compared with PR.

### Absolute values of primary and secondary outcomes

All absolute values (mean ± standard deviation) are shown in Table 2. No significant differences were observed between interventions at baseline (p > 0.05).

**Table 2:** Absolute values about performance, muscle damage, oxidative stress, antioxidant activity, and exertion subjective perception (*n* = 12).

Outcomes	Intervention	Baseline	After the WOD	After intervention	1 hour	24 hours	48 hours
Performance on functional test (counter movement jump test)	PR	43.10 (±5.07)	–	–	40.80 (±3.59)	40.40 (±4.60)	38.86 (±4.69)
	IPC	42.32 (±4.47)	–	–	40.76 (±3.10)	40.81 (±3.87)	40.24 (±2.83)
	EWST	42.61 (±3.07)	–	–	40.36 (±2.04)	41.11 (±3.07)	40.50 (±3.28)
	PBMT-sMF	41.60 (±2.83)	–	–	41.71 (±3.13)	41.46 (±3.99)	41.79 (±3.68)
Muscle damage – LDH (U.L^-1^)	PR	180.34 (±32.71)	–	–	241.87 (±48.58)	306.07 (±58.29)	469.15 (±89.78)
	IPC	167.45 (±43.43)	–	–	243.58 (±35.68)	303.15 (±50.73)	454.30 (±60.69)
	EWST	179.43 (±55.36)	–	–	237.02 (±48.41)	300.78 (±49.04)	456.49 (±72.38)
	PBMT-sMF	197.79 (±33.83)	–	–	223.31 (±45.39)	250.81 (±37.10)	412.89 (±68.10)
Oxidative stress – TBARS (nmol/ml)	PR	3.11 (±0.22)	–	–	4.34 (±0.26)	5.55 (±0.45)^****^	6.74 (0.31)^****^
	IPC	3.29 (±0.16)	–	–	4.47 (±0.36)	5.55 (±0.35)^****^	6.85 (0.36)^****^
	ESWT	3.35 (±0.22)	–	–	4.45 (±0.43)	5.70 (±0.55)^****^	7.00 (±0.44)^****^
	PBMT-sMF	3.37 (±0.36)	–	–	4.50 (±0.39)	4.46 (±0.22)	4.65 (±0.23)
Oxidative stress – carbonylated protein (nmol of DPNH/g/DL of protein)	PR	4.90 (±0.45)	–	–	5.87 (±0.50)	6.33 (±0.81)^*^	6.61 (±0.59)^**^
	IPC	5.14 (±0.34)	–	–	5.85 (±0.55)	6.39 (±0.63)^**^	6.48 (±0.74)^*^
	ESWT	5.11 (0.48)	–	–	6.02 (±0.37)	6.36 (±0.61)^**^	6.99 (±0.64)^***^
	PBMT-sMF	5.17 (±0.35)	–	–	5.73 (±0.41)	5.43 (±0.51)	5.60 (±0.64)
Antioxidant activity – CAT (U CAT/g of protein)	PR	4.50 (±0.60)	–	–	4.51 (±0.37)^*^	3.73 (±0.37)^****^	3.34 (±0.25)^****^
	IPC	4.47 (±0.45)	–	–	4.71 (±0.43)	3.53 (±0.32)^****^	3.26 (±0.44)^****^
	ESWT	4.27 (±0.38)	–	–	4.69 (±0.48)	3.61 (±0.45)^****^	3.44 (±0.47)^****^
	PBMT-sMF	4.26 (±0.37)	–	–	4.98 (±0.39)	5.32 (±0.38)	5.11 (±0.39)
Antioxidant activity – SOD (U SOD/g of protein)	PR	3.53 (±0.33)	–	–	3.40 (±0.43)^*^	3.27 (±0.45)^****^	2.95 (±0.38)^****^
	IPC	3.47 (±0.35)	–	–	3.52 (±0.42)	3.00 (±0.46)^****^	2.92 (±0.39)^****^
	ESWT	3.39 (±0.29)	–	–	3.60 (±0.33)	3.24 (±0.43)^****^	3.07 (±0.44)^****^
	PBMT-sMF	3.42 (±0.43)	–	–	3.91 (±0.41)	4.25 (±0.37)	4.40 (±0.40)
Exertion subjective perception RPE – R (0 −100)	PR	4.75 (±9.53)	78.42 (±23.06)	5.42 (±8.91)	4.67 (±8.23)	1.38 (±3.01)	0.25 (±0.87)
	IPC	0.50 (±1.45)	84.42 (±15.27)	10.42 (±16.44)	9.00 (±16.90)	0.83 (±2.89)	0.50 (±1.73)
	ESWT	0.29 (±0.69)	78.33 (±23.00)	3.08 (±4.81)	1.67 (±3.26)	1.00 (±3.46)	0.83 (±2.89)
	PBMT-sMF	3.33 (±6.85)	78.58 (±25.96)	5.25 (±7.50)	1.83 (±4.41)	0.42 (±1.44)	0.00 (±0.00)
Exertion subjective perception RPE – MI (0–100)	PR	6.71 (±7.89)	72.92 (±24.42)	32.50 (±20.06)	29.67 (±22.46)	21.83 (21.66)	15.17 (18.08)
	IPC	13.17 (±13.10)	81.67 (±19.11)	29.58 (±18.88)	27.50 (±18.21)	19.13 (±15.06)	11.13 (±14.24)
	ESWT	14.50 (±19.64)	82.92 (±16.16)	32.67 (±21.82)	27.58 (±25.84)	14.58 (±15.28)	12.83 (±10.83)
	PBMT-sMF	20.33 (±16.23)	85.17 (±13.83)	34.83 (±12.21)	29.33 (±16.70)	20.67 (±15.60)	14.58 (±12.67)

Values are expressed as mean ± standard deviation (SD). Comparisons of PR, IPC, and ESWT versus PBMT-sMF: *p < 0.05; **p < 0.01; ***p < 0.001; ****p < 0.0001. Statistical analysis was performed using ANOVA with baseline adjustment.

## Discussion

This study is the first to compare the effects of different therapeutic modalities on recovery following a WOD in CrossFit athletes, with an emphasis on reducing muscle damage and enhancing functional performance. We observed that there was no significant difference between interventions in vertical jump performance at 1-hour post-WOD. However, for the secondary outcomes, we observed that PBMT-sMF demonstrated greater improvements in vertical jump performance compared with PR, and outperformed all other therapies in reducing muscle damage and modulating oxidative stress.

The lack of differences in CMJ height at 1-hour post-WOD may be related to the marked neuromuscular fatigue that follows high-intensity functional exercise. In this early recovery window, metabolic disturbances, reduced motor coordination, and greater variability in movement strategy may obscure subtle intervention effects [33,34]. In addition, although the CMJ was assessed using a validated smartphone application, this method may introduce small measurement variations compared with laboratory-grade equipment, potentially increasing variability at this early time point. CMJ height is also less sensitive to treatment effects immediately after exercise, as early impairments are strongly influenced by phosphocreatine depletion, metabolic acidosis, and transient neural inhibition [35,36]. Moreover, the physiological mechanisms associated with PBMT-sMF typically manifest later in recovery, which aligns with the improvements observed at 24 and 48 hours [5]. Finally, there is no scientific evidence supporting positive effects of either IPC or ESWT on post-exercise recovery, muscle performance, or muscle damage reduction, regardless of the recovery time window assessed [20,24,37–41].

Given this physiological context, it is important to note that CrossFit WODs are characterised by repeated high-intensity, multimodal efforts that impose substantial mechanical and metabolic stress. Such sessions commonly lead to marked elevations in muscle damage and oxidative stress biomarkers, including CK, LDH, and indices of redox imbalance [42,43]. These well-documented responses help contextualise our findings, as the reductions in muscle damage and the modulation of oxidative stress observed following PBMT-sMF are consistent with the physiological disturbances typically induced by high-intensity functional training.

Several studies have previously investigated the effects of PBMT and PBMT-sMF on post-exercise recovery, with most reporting findings consistent with our results. These studies demonstrated that PBMT and PBMT-sMF contribute to improved muscle performance, reduced muscle damage, oxidative stress modulation, and enhanced antioxidant activity [4,15,16,18,28,44–49]. However, methodological differences exist among these trials. Various exercise protocols have been employed, including concentric and eccentric isokinetic contractions, isometric contractions, cardiopulmonary and plyometric exercises, and sport-specific performance tests. Additionally, different variables have been analysed as primary outcomes, such as time to exhaustion or number of repetitions for functional performance assessment, while muscle damage has been evaluated through CK and LDH levels. Importantly, only a few studies have investigated the effects of PBMT and PBMT-sMF on markers of oxidative stress to better understand their potential role in enhancing post-exercise recovery and performance [50]. Moreover, most trials compared PBMT or PBMT-sMF to a placebo, whereas non-placebo-controlled studies primarily compared PBMT to cryotherapy [18,44]. Our study is the first to compare PBMT-sMF with three different therapeutic modalities for post-exercise recovery. A systematic review has previously confirmed the efficacy of PBMT and PBMT-sMF in improving muscle performance, reducing muscle damage, and accelerating recovery, despite methodological variations among studies [5]. Another systematic review provided evidence supporting the ability of PBMT and PBMT-sMF to modulate oxidative stress and enhance antioxidant activity [50]. These findings are consistent with our results, reinforcing the superiority of PBMT-sMF over other therapeutic modalities in enhancing functional performance and reducing muscle damage after fatiguing exercise, likely through mechanisms involving the modulation of inflammatory and oxidative stress markers.

Evidence regarding IPC’s effects on post-exercise recovery remains limited. Existing trials have reported results similar to ours, indicating that IPC is not superior to placebo or other recovery methods [20,37–41]. Methodological differences exist between these studies and our trial. While our study focused on CrossFit athletes, previous studies examined the effects of IPC in runners, rugby players, and endurance-trained individuals. These populations differ from CrossFit athletes in training structure, intensity, and physiological demands, which may influence recovery responses. Additionally, different variables were assessed, including CK levels, muscle fatigue (via visual analog scale), blood lactate, CRP, dynamometry, soreness, flexibility, biopsies, pain, and range of motion. Furthermore, some trials administered multiple IPC sessions post-exercise [37–39]. In comparative analysis, IPC has been tested against passive recovery, massage, intermittent negative pressure, placebo, or sham treatments, but not against electrophysical agents such as PBMT or PBMT-sMF. Consistent with our findings, no previous studies have demonstrated IPC’s effectiveness in post-exercise recovery, muscle performance improvement, or muscle damage reduction [20,37–41]. Furthermore, a comparison of our results with earlier research regarding oxidative stress is not possible, as none of these studies investigated the effects of IPC on this outcome.

Although ESWT has been increasingly used in sports recovery, there is currently no strong scientific basis supporting its clinical application. To our knowledge, only one trial has examined the effects of a single ESWT session on eccentric exercise-induced delayed-onset muscle soreness (DOMS) [24]. This study included healthy adults who received either ESWT, placebo, or no treatment. The outcomes assessed were pain intensity, maximum voluntary isometric force, pressure pain threshold, and impairment in daily activities. The results indicated that ESWT was ineffective for DOMS. While these findings align with ours, showing that ESWT was not superior to other treatments or no treatment, methodological differences between the studies—particularly regarding the outcomes assessed—make direct comparisons difficult.

Our findings highlight the superiority of PBMT-sMF over PR, IPC, and ESWT, with no reported adverse effects, consistent with previous observations [51]. Recent discussions have suggested that modulation of oxidative stress and enhancement of antioxidant activity may underlie the beneficial effects of PBMT-sMF in improving performance and expediting post-exercise recovery. Our results support this hypothesis, indicating that PBMT-sMF enhances antioxidant defences, thereby mitigating exercise-induced oxidative stress. In contrast, the other modalities tested did not demonstrate effectiveness in improving performance or enhancing recovery, nor were they effective in reducing muscle damage or modulating oxidative stress markers. Therefore, although IPC and ESWT are commonly used in clinical practice, our findings do not support their efficacy. The only outcome in which IPC and ESWT outperformed both passive recovery and PBMT-sMF was participant satisfaction. This pattern may reflect placebo-related influences, sensory stimulation, or expectations of benefit associated with device-based treatments. These perceptions may still play a role in how athletes experience recovery, which is worth considering in clinical contexts. Conversely, our study provides evidence supporting PBMT-sMF as an effective strategy to improve performance, minimize muscle damage, and accelerate recovery after high-intensity exercise. Our findings are generalizable only to non-professional male CrossFit athletes.

One of the strengths of this study is that the sample size was sufficient to detect the observed effect size for the primary outcome, as confirmed by a post hoc power analysis. Additionally, this trial was prospectively registered, and methodological rigor was ensured through allocation concealment and an intention-to-treat analysis. Notably, no participants dropped out during the study, and no learning effects were observed, suggesting that the observed effects were attributable to the tested interventions. However, some limitations should be acknowledged. First, the study included only male participants, limiting the generalizability of the findings to female athletes, who may respond differently to both the exercise stimulus and the recovery interventions. Additionally, due to the nature of the interventions, blinding was only possible for outcome assessors. Moreover, although the CMJ was assessed using a validated smartphone application, this method may offer slightly lower precision than laboratory-grade equipment. Furthermore, although the sample size was determined to adequately detect differences in the primary outcome, it may have offered more limited power for some of the secondary outcomes, particularly those with greater variability. Finally, the absence of a placebo or sham condition for the active interventions represents an additional methodological limitation, particularly regarding the ability to isolate potential placebo- or expectation-related effects. While passive recovery allowed comparison against a no-treatment condition, it did not control for the perceptual or sensory experiences associated with receiving an intervention. Nonetheless, the consistency of the physiological and functional patterns observed, together with the magnitude of the effects detected, supports the relevance of our findings.

Further studies with robust methodological designs and adequately powered samples are needed to compare IPC and ESWT with other therapeutic modalities, such as cryotherapy, cold water immersion, and massage. Future studies may also benefit from prioritizing primary outcomes assessed at 24 hours or later, as this timeframe appears more suitable for capturing meaningful recovery-related changes following high-intensity exercise. In addition, future research should also include dedicated power analyses for secondary outcomes and consider alternative strategies for handling multiple comparisons when appropriate. Additionally, longer experimental time points, multiple treatment sessions per intervention, and the inclusion of larger and more diverse samples, including female athletes, are recommended to enhance external validity, enable subgroup analyses, and improve the generalizability of the findings.

## Conclusion

PBMT-sMF appears to be an effective and safe recovery strategy for high-intensity workouts such as CrossFit, improving performance and recovery, likely through the mitigation of muscle damage and oxidative stress. Coaches and practitioners may consider its use to enhance post-exercise recovery. IPC and ESWT did not show clear benefits over passive recovery.

## Supporting information

S1 Fig Summary of study procedures and assessments. This figure provides an overview of all procedures and assessments conducted during the study, including their timing and sequence.
(TIFF)

S1 Table Results of sphericity testing and detailed statistical comparisons between interventions. This table presents the results of Mauchly’s test of sphericity, including epsilon values and corrected degrees of freedom used in the repeated-measures ANOVA, as well as detailed between-intervention comparisons, including mean differences, 95% confidence intervals, p-values, and effect sizes.
(PDF)

S2 Table Detailed statistical comparisons between interventions. This table presents detailed results of between-intervention comparisons, including mean differences, 95% confidence intervals, p-values, and effect sizes. (PDF)

S1 Dataset Complete dataset underlying the findings of this study. This file contains all individual data points used in the analyses, including the data underlying all summary statistics presented in the manuscript.
(DOCX)
