# Supplementary figures and images for: A randomised crossover trial comparing photobiomodulation therapy with other recovery strategies in CrossFit athletes
Source: PLoS One. 2026 May 22;21(5):e0349880. doi: 10.1371/journal.pone.0349880 (PMC13196929; doi:10.1371/journal.pone.0349880)

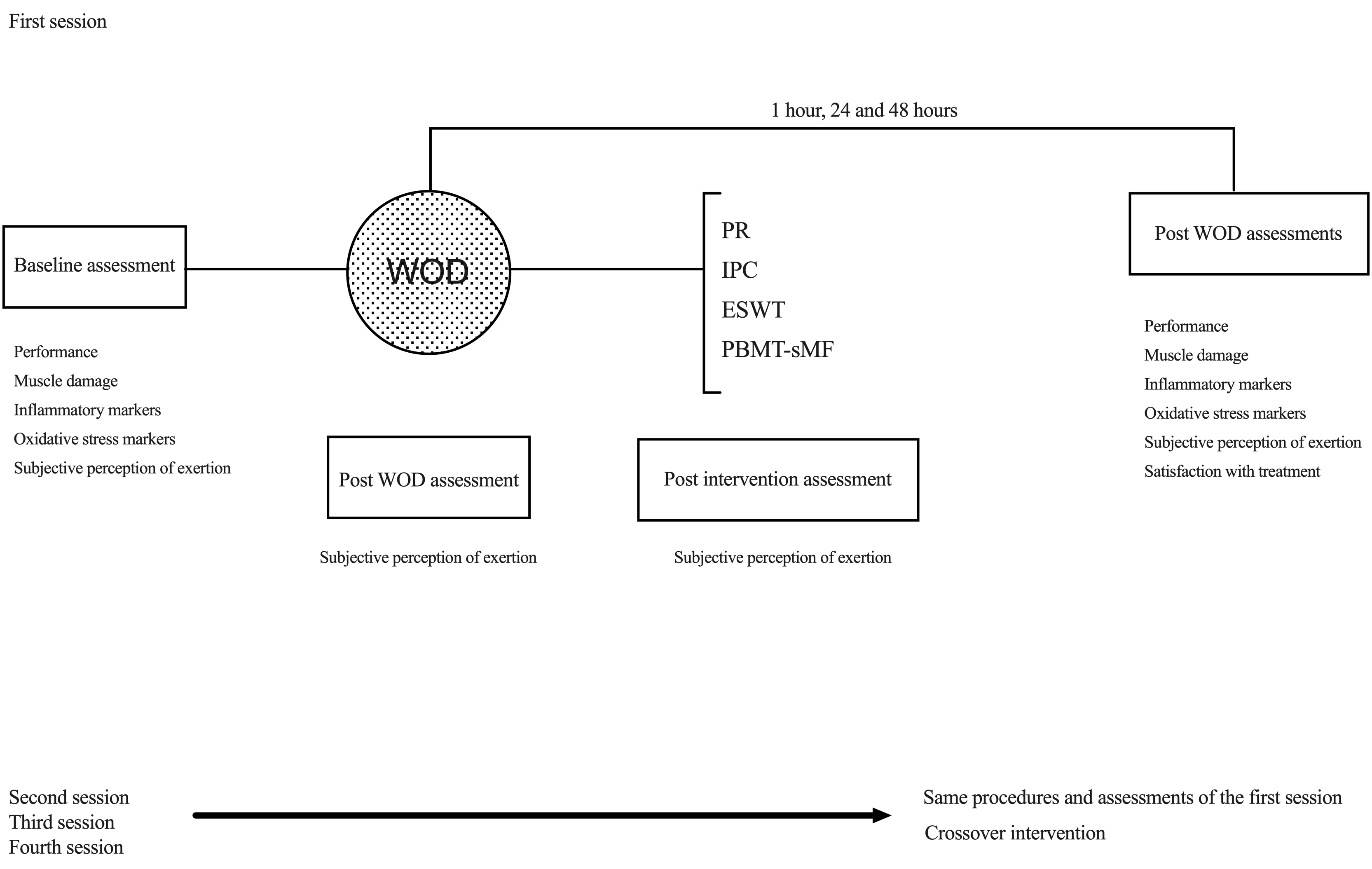

Supplement: S1 Fig — This figure provides an overview of all procedures and assessments conducted during the study, including their timing and sequence. (TIFF) [file pone.0349880.s001.tiff]
