# Supplementary material for: A randomised crossover trial comparing photobiomodulation therapy with other recovery strategies in CrossFit athletes
Source: PLoS One. 2026 May 22;21(5):e0349880. doi: 10.1371/journal.pone.0349880 (PMC13196929; doi:10.1371/journal.pone.0349880)
Supplement: S1 Table — This table presents the results of Mauchly’s test of sphericity, including epsilon values and corrected degrees of freedom used in the repeated-measures ANOVA, as well as detailed between-intervention comparisons, including mean differences, 95% confidence intervals, p-values, and effect sizes. (PDF) [file pone.0349880.s002.pdf]

2way ANOVA
ANOVA results

1	Table Analyzed	CMJ - Absolute				
2						
3	**Two-way RM ANOVA**	Matching: Stacked				
4	Assume sphericity?	No				
5	Alpha	0.05				
6						
7	**Source of Variation**	**% of total variation**	**P value**	**P value summary**	**Significant?**	**Geisser-Greenhouse's epsilon**
8	Time x Treatment	2.595	0.0580	ns	No	0.8683
9	Time	4.344	<0.0001	****	Yes	0.8683
10	Treatment	0.7189	0.9327	ns	No	
11	Subject	73.01	<0.0001	****	Yes	
12						
13	**ANOVA table**	**SS**	**DF**	**MS**	**F (DFn, DFd)**	**P value**
14	Time x Treatment	66.54	9	7.393	F (7.815, 114.6) = 1.969	P=0.0580
15	Time	111.4	3	37.13	F (2.605, 114.6) = 9.888	P<0.0001
16	Treatment	18.44	3	6.146	F (3, 44) = 0.1444	P=0.9327
17	Subject	1872	44	42.55	F (44, 132) = 11.33	P<0.0001
18	Residual	495.7	132	3.755		
19						
20	**Data summary**					
21	Number of columns (Treatment)	4				
22	Number of rows (Time)	4				
23	Number of subjects (Subject)	48				
24	Number of missing values	0				

2way ANOVA
ANOVA results

1	Table Analyzed	CMJ - Change in %				
2						
3	**Two-way RM ANOVA**	Matching: Stacked				
4	Assume sphericity?	No				
5	Alpha	0.05				
6						
7	**Source of Variation**	**% of total variation**	**P value**	**P value summary**	**Significant?**	**Geisser-Greenhouse's epsilon**
8	Time x Treatment	5.491	0.0558	ns	No	0.8570
9	Time	8.618	<0.0001	****	Yes	0.8570
10	Treatment	10.91	0.0067	**	Yes	
11	Subject	34.57	<0.0001	****	Yes	
12						
13	**ANOVA table**	**SS**	**DF**	**MS**	**F (DFn, DFd)**	**P value**
14	Time x Treatment	353.9	9	39.32	F (7.713, 113.1) = 1.993	P=0.0558
15	Time	555.5	3	185.2	F (2.571, 113.1) = 9.385	P<0.0001
16	Treatment	703.4	3	234.5	F (3, 44) = 4.630	P=0.0067
17	Subject	2228	44	50.64	F (44, 132) = 2.567	P<0.0001
18	Residual	2604	132	19.73		
19						
20	**Data summary**					
21	Number of columns (Treatment)	4				
22	Number of rows (Time)	4				
23	Number of subjects (Subject)	48				
24	Number of missing values	0				

2way ANOVA
ANOVA results

1	Table Analyzed	Muscular effort - Absolute				
2						
3	**Two-way RM ANOVA**	Matching: Stacked				
4	Assume sphericity?	No				
5	Alpha	0.05				
6						
7	**Source of Variation**	**% of total variation**	**P value**	**P value summary**	**Significant?**	**Geisser-Greenhouse's epsilon**
8	Time x Treatment	0.8488	0.6490	ns	No	0.5955
9	Time	64.63	<0.0001	****	Yes	0.5955
10	Treatment	0.3449	0.8374	ns	No	
11	Subject	17.88	<0.0001	****	Yes	
12						
13	**ANOVA table**	**SS**	**DF**	**MS**	**F (DFn, DFd)**	**P value**
14	Time x Treatment	2034	15	135.6	F (8.933, 131.0) = 0.7638	P=0.6490
15	Time	154849	5	30970	F (2.978, 131.0) = 174.5	P<0.0001
16	Treatment	826.5	3	275.5	F (3, 44) = 0.2830	P=0.8374
17	Subject	42839	44	973.6	F (44, 220) = 5.485	P<0.0001
18	Residual	39050	220	177.5		
19						
20	**Data summary**					
21	Number of columns (Treatment	4				
22	Number of rows (Time)	6				
23	Number of subjects (Subject)	48				
24	Number of missing values	0				

2way ANOVA
ANOVA results

1	Table Analyzed	Respiratory effort - Absolute				
2						
3	**Two-way RM ANOVA**	Matching: Stacked				
4	Assume sphericity?	No				
5	Alpha	0.05				
6						
7	**Source of Variation**	**% of total variation**	**P value**	**P value summary**	**Significant?**	**Geisser-Greenhouse's epsilon**
8	Time x Treatment	0.2941	0.7761	ns	No	0.3268
9	Time	87.60	<0.0001	****	Yes	0.3268
10	Treatment	0.1721	0.5081	ns	No	
11	Subject	3.210	0.0023	**	Yes	
12						
13	**ANOVA table**	**SS**	**DF**	**MS**	**F (DFn, DFd)**	**P value**
14	Time x Treatment	803.3	15	53.55	F (4.902, 71.89) = 0.4942	P=0.7761
15	Time	239265	5	47853	F (1.634, 71.89) = 441.6	P<0.0001
16	Treatment	470.0	3	156.7	F (3, 44) = 0.7862	P=0.5081
17	Subject	8768	44	199.3	F (44, 220) = 1.839	P=0.0023
18	Residual	23837	220	108.4		
19						
20	**Data summary**					
21	Number of columns (Treatment	4				
22	Number of rows (Time)	6				
23	Number of subjects (Subject)	48				
24	Number of missing values	0				

2way ANOVA
ANOVA results

1	Table Analyzed	LDH - Absolute				
2						
3	**Two-way RM ANOVA**	Matching: Stacked				
4	Assume sphericity?	No				
5	Alpha	0.05				
6						
7	**Source of Variation**	**% of total variation**	**P value**	**P value summary**	**Significant?**	**Geisser-Greenhouse's epsilon**
8	Time x Treatment	1.349	0.0489	*	Yes	0.7816
9	Time	77.06	<0.0001	****	Yes	0.7816
10	Treatment	0.8798	0.3432	ns	No	
11	Subject	11.31	<0.0001	****	Yes	
12						
13	**ANOVA table**	**SS**	**DF**	**MS**	**F (DFn, DFd)**	**P value**
14	Time x Treatment	33386	9	3710	F (7.035, 103.2) = 2.107	P=0.0489
15	Time	1906577	3	635526	F (2.345, 103.2) = 360.9	P<0.0001
16	Treatment	21766	3	7255	F (3, 44) = 1.141	P=0.3432
17	Subject	279892	44	6361	F (44, 132) = 3.613	P<0.0001
18	Residual	232432	132	1761		
19						
20	**Data summary**					
21	Number of columns (Treatment	4				
22	Number of rows (Time)	4				
23	Number of subjects (Subject)	48				
24	Number of missing values	0				

	2way ANOVA<br>ANOVA results					
1	Table Analyzed	LDH - Change in %				
2						
3	**Two-way RM ANOVA**	Matching: Stacked				
4	Assume sphericity?	No				
5	Alpha	0.05				
6						
7	**Source of Variation**	**% of total variation**	**P value**	**P value summary**	**Significant?**	**Geisser-Greenhouse's epsilon**
8	Time x Treatment	2.249	0.0074	**	Yes	0.7658
9	Time	69.83	<0.0001	****	Yes	0.7658
10	Treatment	4.964	0.0014	**	Yes	
11	Subject	11.85	<0.0001	****	Yes	
12						
13	**ANOVA table**	**SS**	**DF**	**MS**	**F (DFn, DFd)**	**P value**
14	Time x Treatment	20905	9	2323	F (6.893, 101.1) = 2.971	P=0.0074
15	Time	648937	3	216312	F (2.298, 101.1) = 276.7	P<0.0001
16	Treatment	46137	3	15379	F (3, 44) = 6.142	P=0.0014
17	Subject	110164	44	2504	F (44, 132) = 3.202	P<0.0001
18	Residual	103200	132	781.8		
19						
20	**Data summary**					
21	Number of columns (Treatment	4				
22	Number of rows (Time)	4				
23	Number of subjects (Subject)	48				
24	Number of missing values	0				

2way ANOVA
ANOVA results

1	Table Analyzed	TBARS - Absolute				
2						
3	**Two-way RM ANOVA**	Matching: Stacked				
4	Assume sphericity?	No				
5	Alpha	0.05				
6						
7	**Source of Variation**	**% of total variation**	**P value**	**P value summary**	**Significant?**	**Geisser-Greenhouse's epsilon**
8	Time x Treatment	10.64	<0.0001	****	Yes	0.8764
9	Time	75.29	<0.0001	****	Yes	0.8764
10	Treatment	7.371	<0.0001	****	Yes	
11	Subject	1.392	0.8188	ns	No	
12						
13	**ANOVA table**	**SS**	**DF**	**MS**	**F (DFn, DFd)**	**P value**
14	Time x Treatment	33.74	9	3.749	F (7.888, 115.7) = 29.40	P<0.0001
15	Time	238.7	3	79.57	F (2.629, 115.7) = 624.0	P<0.0001
16	Treatment	23.37	3	7.790	F (3, 44) = 77.66	P<0.0001
17	Subject	4.413	44	0.1003	F (44, 132) = 0.7867	P=0.8188
18	Residual	16.83	132	0.1275		
19						
20	**Data summary**					
21	Number of columns (Treatment)	4				
22	Number of rows (Time)	4				
23	Number of subjects (Subject)	48				
24	Number of missing values	0				

2way ANOVA
ANOVA results

1	Table Analyzed	TBARS - Change in %				
2						
3	**Two-way RM ANOVA**	Matching: Stacked				
4	Assume sphericity?	No				
5	Alpha	0.05				
6						
7	**Source of Variation**	**% of total variation**	**P value**	**P value summary**	**Significant?**	**Geisser-Greenhouse's epsilon**
8	Time x Treatment	10.17	<0.0001	****	Yes	0.9739
9	Time	69.46	<0.0001	****	Yes	0.9739
10	Treatment	8.912	<0.0001	****	Yes	
11	Subject	5.616	<0.0001	****	Yes	
12						
13	**ANOVA table**	**SS**	**DF**	**MS**	**F (DFn, DFd)**	**P value**
14	Time x Treatment	32412	9	3601	F (8.765, 128.6) = 25.56	P<0.0001
15	Time	221283	3	73761	F (2.922, 128.6) = 523.5	P<0.0001
16	Treatment	28393	3	9464	F (3, 44) = 23.27	P<0.0001
17	Subject	17893	44	406.7	F (44, 132) = 2.886	P<0.0001
18	Residual	18597	132	140.9		
19						
20	**Data summary**					
21	Number of columns (Treatment)	4				
22	Number of rows (Time)	4				
23	Number of subjects (Subject)	48				
24	Number of missing values	0				

2way ANOVA
ANOVA results

1	Table Analyzed	Carbonylated proteins - Absolute				
2						
3	**Two-way RM ANOVA**	Matching: Stacked				
4	Assume sphericity?	No				
5	Alpha	0.05				
6						
7	**Source of Variation**	**% of total variation**	**P value**	**P value summary**	**Significant?**	**Geisser-Greenhouse's epsilon**
8	Time x Treatment	8.536	0.0011	**	Yes	0.9065
9	Time	38.78	<0.0001	****	Yes	0.9065
10	Treatment	8.700	<0.0001	****	Yes	
11	Subject	8.128	0.9289	ns	No	
12						
13	**ANOVA table**	**SS**	**DF**	**MS**	**F (DFn, DFd)**	**P value**
14	Time x Treatment	10.49	9	1.165	F (8.158, 119.7) = 3.492	P=0.0011
15	Time	47.64	3	15.88	F (2.719, 119.7) = 47.59	P<0.0001
16	Treatment	10.69	3	3.562	F (3, 44) = 15.70	P<0.0001
17	Subject	9.985	44	0.2269	F (44, 132) = 0.6801	P=0.9289
18	Residual	44.04	132	0.3336		
19						
20	**Data summary**					
21	Number of columns (Treatment)	4				
22	Number of rows (Time)	4				
23	Number of subjects (Subject)	48				
24	Number of missing values	0				

2way ANOVA
ANOVA results

1	Table Analyzed	Carbonylated proteins - Change in %				
2						
3	**Two-way RM ANOVA**	Matching: Stacked				
4	Assume sphericity?	No				
5	Alpha	0.05				
6						
7	**Source of Variation**	**% of total variation**	**P value**	**P value summary**	**Significant?**	**Geisser-Greenhouse's epsilon**
8	Time x Treatment	5.866	0.0019	**	Yes	0.9227
9	Time	28.34	<0.0001	****	Yes	0.9227
10	Treatment	9.669	0.0057	**	Yes	
11	Subject	29.69	<0.0001	****	Yes	
12						
13	**ANOVA table**	**SS**	**DF**	**MS**	**F (DFn, DFd)**	**P value**
14	Time x Treatment	4176	9	464.0	F (8.305, 121.8) = 3.255	P=0.0019
15	Time	20180	3	6727	F (2.768, 121.8) = 47.19	P<0.0001
16	Treatment	6884	3	2295	F (3, 44) = 4.777	P=0.0057
17	Subject	21138	44	480.4	F (44, 132) = 3.370	P<0.0001
18	Residual	18818	132	142.6		
19						
20	**Data summary**					
21	Number of columns (Treatment)	4				
22	Number of rows (Time)	4				
23	Number of subjects (Subject)	48				
24	Number of missing values	0				

2way ANOVA
ANOVA results

1	Table Analyzed	CAT - Absolute				
2						
3	**Two-way RM ANOVA**	Matching: Stacked				
4	Assume sphericity?	No				
5	Alpha	0.05				
6						
7	**Source of Variation**	**% of total variation**	**P value**	**P value summary**	**Significant?**	**Geisser-Greenhouse's epsilon**
8	Time x Treatment	23.79	<0.0001	****	Yes	0.8871
9	Time	21.45	<0.0001	****	Yes	0.8871
10	Treatment	27.09	<0.0001	****	Yes	
11	Subject	6.312	0.6703	ns	No	
12						
13	**ANOVA table**	**SS**	**DF**	**MS**	**F (DFn, DFd)**	**P value**
14	Time x Treatment	26.26	9	2.917	F (7.984, 117.1) = 16.34	P<0.0001
15	Time	23.68	3	7.892	F (2.661, 117.1) = 44.20	P<0.0001
16	Treatment	29.90	3	9.967	F (3, 44) = 62.95	P<0.0001
17	Subject	6.967	44	0.1583	F (44, 132) = 0.8867	P=0.6703
18	Residual	23.57	132	0.1786		
19						
20	**Data summary**					
21	Number of columns (Treatment)	4				
22	Number of rows (Time)	4				
23	Number of subjects (Subject)	48				
24	Number of missing values	0				

2way ANOVA
ANOVA results

1	Table Analyzed	CAT - Change in %				
2						
3	**Two-way RM ANOVA**	Matching: Stacked				
4	Assume sphericity?	No				
5	Alpha	0.05				
6						
7	**Source of Variation**	**% of total variation**	**P value**	**P value summary**	**Significant?**	**Geisser-Greenhouse's epsilon**
8	Time x Treatment	17.89	<0.0001	****	Yes	0.8605
9	Time	15.72	<0.0001	****	Yes	0.8605
10	Treatment	28.41	<0.0001	****	Yes	
11	Subject	21.26	<0.0001	****	Yes	
12						
13	**ANOVA table**	**SS**	**DF**	**MS**	**F (DFn, DFd)**	**P value**
14	Time x Treatment	14017	9	1557	F (7.744, 113.6) = 15.69	P<0.0001
15	Time	12320	3	4107	F (2.581, 113.6) = 41.38	P<0.0001
16	Treatment	22263	3	7421	F (3, 44) = 19.60	P<0.0001
17	Subject	16660	44	378.6	F (44, 132) = 3.815	P<0.0001
18	Residual	13100	132	99.24		
19						
20	**Data summary**					
21	Number of columns (Treatment)	4				
22	Number of rows (Time)	4				
23	Number of subjects (Subject)	48				
24	Number of missing values	0				

2way ANOVA
ANOVA results

1	Table Analyzed	SOD - Absolute				
2						
3	**Two-way RM ANOVA**	Matching: Stacked				
4	Assume sphericity?	No				
5	Alpha	0.05				
6						
7	**Source of Variation**	**% of total variation**	**P value**	**P value summary**	**Significant?**	**Geisser-Greenhouse's epsilon**
8	Time x Treatment	20.63	<0.0001	****	Yes	0.9015
9	Time	2.968	0.0105	*	Yes	0.9015
10	Treatment	30.73	<0.0001	****	Yes	
11	Subject	13.86	0.1251	ns	No	
12						
13	**ANOVA table**	**SS**	**DF**	**MS**	**F (DFn, DFd)**	**P value**
14	Time x Treatment	12.56	9	1.395	F (8.114, 119.0) = 9.510	P<0.0001
15	Time	1.807	3	0.6022	F (2.705, 119.0) = 4.105	P=0.0105
16	Treatment	18.70	3	6.234	F (3, 44) = 32.51	P<0.0001
17	Subject	8.439	44	0.1918	F (44, 132) = 1.307	P=0.1251
18	Residual	19.36	132	0.1467		
19						
20	**Data summary**					
21	Number of columns (Treatment)	4				
22	Number of rows (Time)	4				
23	Number of subjects (Subject)	48				
24	Number of missing values	0				

2way ANOVA
ANOVA results

1	Table Analyzed	SOD - Change in %				
2						
3	**Two-way RM ANOVA**	Matching: Stacked				
4	Assume sphericity?	No				
5	Alpha	0.05				
6						
7	**Source of Variation**	**% of total variation**	**P value**	**P value summary**	**Significant?**	**Geisser-Greenhouse's epsilon**
8	Time x Treatment	18.28	<0.0001	****	Yes	0.9192
9	Time	2.443	0.0128	*	Yes	0.9192
10	Treatment	32.18	<0.0001	****	Yes	
11	Subject	19.49	0.0006	***	Yes	
12						
13	**ANOVA table**	**SS**	**DF**	**MS**	**F (DFn, DFd)**	**P value**
14	Time x Treatment	11026	9	1225	F (8.273, 121.3) = 9.709	P<0.0001
15	Time	1474	3	491.2	F (2.758, 121.3) = 3.893	P=0.0128
16	Treatment	19416	3	6472	F (3, 44) = 24.22	P<0.0001
17	Subject	11757	44	267.2	F (44, 132) = 2.118	P=0.0006
18	Residual	16656	132	126.2		
19						
20	**Data summary**					
21	Number of columns (Treatment)	4				
22	Number of rows (Time)	4				
23	Number of subjects (Subject)	48				
24	Number of missing values	0				

Table 1. Between-intervention comparisons of changes in functional test performance across time points.

Time point	Comparisons	Mean difference	95% CI	p- value	Effect Size
Baseline	ESWT vs. PBMT-sMF	0.00	-	-	-
	IPC vs. PBMT-sMF	0.00	-	-	-
	PR vs. PBMT-sMF	0.00	-	-	-
	IPC vs. ESWT	0.00	-	-	-
	PR vs. ESWT	0.00	-	-	-
	PR vs. IPC	0.00	-	-	-
1 hour	ESWT vs. PBMT-sMF	-5.42	-13.64 to 2.79	0.41	0.542
	IPC vs. PBMT-sMF	-3.57	-12.76 to 5.61	>1.00	0.378
	PR vs. PBMT-sMF	-5.38	-13.14 to 2.39	0.34	0.567
	IPC vs. ESWT	1.85	-6.90 to 10.60	>1.00	0.198
	PR vs. ESWT	0.05	-7.12 to 7.21	>1.00	0.008
	PR vs. IPC	-1.80	-10.16 to 6.55	>1.00	0.254
24 hours	ESWT vs. PBMT-sMF	-3.01	-10.47 to 4.44	>1.00	0.272
	IPC vs. PBMT-sMF	-3.06	-9.69 to 3.56	>1.00	0.508
	PR vs. PBMT-sMF	-5.81	-12.28 to 0.64	0.09	0.797
	IPC vs. ESWT	-0.05	-5.99 to 5.89	>1.00	0.006
	PR vs. ESWT	-2.81	-8.54 to 2.93	0.97	0.373
	PR vs. IPC	-2.75	-6.94 to 1.43	0.41	0.616
48 hours	ESWT vs. PBMT-sMF	-5.34	-11.97 to 1.28	0.17	0.560
	IPC vs. PBMT-sMF	-4.87	-12.71 to 2.97	0.50	0.617
	PR vs. PBMT-sMF	-10.16	-16.86 to -3.46	0.00	1.503
	IPC vs. ESWT	0.47	-6.10 to 7.94	>1.00	0.044
	PR vs. ESWT	-4.81	-11.02 to 1.39	0.21	0.734
	PR vs. IPC	-5.28	-12.82 to 2.24	0.32	0.625

Values are presented as mean differences (MD) with 95% confidence intervals (CI). Effect sizes were calculated using Cohen’s d. Time points included baseline. 1 hour. 24 hours. and 48 hours. PR = passive recovery; IPC = intermittent pneumatic compression; ESWT = extracorporeal shockwave therapy; PBMT-sMF = photobiomodulation therapy combined with static magnetic field.

Table 2. Between-intervention comparisons of absolute functional test performance across time points.

Time point	Comparisons	Mean difference	95% CI	p- value	Effect Size
Baseline	ESWT vs. PBMT-sMF	1.01	-2.49 to 4.51	>1.00	0.473
	IPC vs. PBMT-sMF	0.71	-3.80 to 5.22	>1.00	0.239
	PR vs. PBMT-sMF	1.50	-3.50 to 6.49	>1.00	0.427
	IPC vs. ESWT	-0.30	-4.89 to 4.30	>1.00	0.092
	PR vs. ESWT	0.48	-4.58 to 5.55	>1.00	0.133
	PR vs. IPC	0.78	-4.88 to 6.44	>1.00	0.292
1 hour	ESWT vs. PBMT-sMF	-1.35	-4.52 to 1.83	>1.00	0.393
	IPC vs. PBMT-sMF	-0.94	-4.63 to 2.74	>1.00	0.235
	PR vs. PBMT-sMF	-0.91	-4.90 to 3.08	>1.00	0.209
	IPC vs. ESWT	0.40	-2.75 to 3.55	>1.00	0.107
	PR vs. ESWT	0.44	-3.11 to 3.98	>1.00	0.116
	PR vs. IPC	0.037	-3.94 to 4.01	>1.00	0.012
24 hours	ESWT vs. PBMT-sMF	-0.34	-4.58 to 3.89	>1.00	0.099
	IPC vs. PBMT-sMF	-0.65	-5.30 to 4.00	>1.00	0.200
	PR vs. PBMT-sMF	-1.06	-6.16 to 4.04	>1.00	0.240
	IPC vs. ESWT	-0.30	-4.45 to 3.85	>1.00	0.083
	PR vs. ESWT	-0.71	-5.41 to 3.98	>1.00	0.217
	PR vs. IPC	-0.41	-5.45 to 4.63	>1.00	0.146
48 hours	ESWT vs. PBMT-sMF	-1.30	-5.43 to 2.84	>1.00	0.404
	IPC vs. PBMT-sMF	-1.55	-5.46 to 2.36	>1.00	0.494
	PR vs. PBMT-sMF	-2.93	-7.95 to 2.08	0.6209	0.706
	IPC vs. ESWT	-0.26	-3.89 to 3.37	>1.00	0.078
	PR vs. ESWT	-1.64	-6.48 to 3.21	>1.00	0.544
	PR vs. IPC	-1.38	-6.06 to 3.30	>1.00	0.312

Values are presented as mean differences (MD) with 95% confidence intervals (CI). Effect sizes were calculated using Cohen's d. Time points included baseline. 1 hour. 24 hours. and 48 hours. PR = passive recovery; IPC = intermittent pneumatic compression; ESWT = extracorporeal shockwave therapy; PBMT-sMF = photobiomodulation therapy combined with static magnetic field.

Table 3. Between-intervention comparisons of changes in LDH across time points.

Time point	Comparisons	Mean difference	95% CI	p- value	Effect Size
Baseline	ESWT vs. PBMT-sMF	0.00	-	-	-
	IPC vs. PBMT-sMF	0.00	-	-	-
	PR vs. PBMT-sMF	0.00	-	-	-
	IPC vs. ESWT	0.00	-	-	-
	PR vs. ESWT	0.00	-	-	-
	PR vs. IPC	0.00	-	-	-
1 hour	ESWT vs. PBMT-sMF	23.50	-11.56 to 58.57	0.39	0.628
	IPC vs. PBMT-sMF	34.90	4.40 to 65.40	0.02	0.854
	PR vs. PBMT-sMF	22.36	-16.21 to 60.93	0.63	0.456
	IPC vs. ESWT	11.39	-22.69 to 45.47	>1.00	0.346
	PR vs. ESWT	-1.14	-42.18 to 39.89	>1.00	0.021
	PR vs. IPC	-12.54	-50.29 to 25.21	>1.00	0.244
24 hours	ESWT vs. PBMT-sMF	47.77	11.31 to 84.24	0.01	0.976
	IPC vs. PBMT-sMF	59.14	16.26 to 102.00	0.00	1.596
	PR vs. PBMT-sMF	44.20	5.67 to 82.73	0.02	0.946
	IPC vs. ESWT	11.37	-36.86 to 59.59	>1.00	0.193
	PR vs. ESWT	-3.58	-48.40 to 41.24	>1.00	0.056
	PR vs. IPC	-14.94	-64.45 to 34.57	>1.00	0.229
48 hours	ESWT vs. PBMT-sMF	56.73	3.59 to 109.90	0.03	0.984
	IPC vs. PBMT-sMF	70.29	20.48 to 120.10	0.00	1.825
	PR vs. PBMT-sMF	56.08	-4.25 to 116.40	0.08	0.751
	IPC vs. ESWT	13.57	-49.42 to 76.55	>1.00	0.186
	PR vs. ESWT	-0.65	-71.05 to 69.75	>1.00	0.007
	PR vs. IPC	-14.22	-82.69 to 54.25	>1.00	0.163

Values are presented as mean differences (MD) with 95% confidence intervals (CI). Effect sizes were calculated using Cohen’s d. Time points included baseline. 1 hour. 24 hours. and 48 hours. PR = passive recovery; IPC = intermittent pneumatic compression; ESWT = extracorporeal shockwave therapy; PBMT-sMF = photobiomodulation therapy combined with static magnetic field.

Table 4. Between-intervention comparisons of absolute LDH values across time points.

Time point	Comparisons	Mean difference	95% CI	p- value	Effect Size
Baseline	ESWT vs. PBMT-sMF	-18.36	-73.78 to 37.05	>1.00	0.257
	IPC vs. PBMT-sMF	-30.34	-76.67 to 16.00	0.42	0.603
	PR vs. PBMT-sMF	-17.45	-56.83 to 21.92	>1.00	0.458
	IPC vs. ESWT	-11.97	-71.18 to 47.23	>1.00	0.243
	PR vs. ESWT	0.91	-54.15 to 55.97	>1.00	0.012
	PR vs. IPC	12.88	-32.95 to 58.72	>1.00	0.216
1 hour	ESWT vs. PBMT-sMF	13.71	-41.84 to 69.26	>1.00	0.256
	IPC vs. PBMT-sMF	20.26	-28.31 to 68.83	>1.00	0.420
	PR vs. PBMT-sMF	18.56	-37.09 to 74.21	>1.00	0.332
	IPC vs. ESWT	6.55	-44.20 to 57.31	>1.00	0.110
	PR vs. ESWT	4.84	-52.54 to 62.23	>1.00	0.067
	PR vs. IPC	-1.71	-52.58 to 49.17	>1.00	0.027
24 hours	ESWT vs. PBMT-sMF	49.97	-1.86 to 101.8	0.06	0.829
	IPC vs. PBMT-sMF	52.34	-0.73 to 105.40	0.05	0.803
	PR vs. PBMT-sMF	55.26	-3.58 to 114.10	0.07	0.913
	IPC vs. ESWT	2.37	-56.68 to 61.41	>1.00	0.042
	PR vs. ESWT	5.29	-58.63 to 69.21	>1.00	0.072
	PR vs. IPC	2.92	-61.85 to 67.70	>1.00	0.039
48 hours	ESWT vs. PBMT-sMF	43.60	-39.58 to 126.80	0.86	0.397
	IPC vs. PBMT-sMF	41.41	-35.02 to 117.80	0.78	0.542
	PR vs. PBMT-sMF	56.26	-38.70 to 151.20	0.59	0.613
	IPC vs. ESWT	-2.19	-81.46 to 77.08	>1.00	0.026
	PR vs. ESWT	12.66	-84.26 to 109.60	>1.00	0.099
	PR vs. IPC	14.85	-77.07 to 106.80	>1.00	0.181

Values are presented as mean differences (MD) with 95% confidence intervals (CI). Effect sizes were calculated using Cohen's d. Time points included baseline. 1 hour. 24 hours. and 48 hours. PR = passive recovery; IPC = intermittent pneumatic compression; ESWT = extracorporeal shockwave therapy; PBMT-sMF = photobiomodulation therapy combined with static magnetic field.

Table 5. Between-intervention comparisons of changes in TBARS across time points.

Time point	Comparisons	Mean difference	95% CI	p- value	Effect Size
Baseline	ESWT vs. PBMT-sMF	0.00	-	-	-
	IPC vs. PBMT-sMF	0.00	-	-	-
	PR vs. PBMT-sMF	0.00	-	-	-
	IPC vs. ESWT	0.00	-	-	-
	PR vs. ESWT	0.00	-	-	-
	PR vs. IPC	0.00	-	-	-
1 hour	ESWT vs. PBMT-sMF	-5.99	-27.93 to 15.96	>1.00	0.097
	IPC vs. PBMT-sMF	0.61	-19.51 to 20.73	>1.00	0.037
	PR vs. PBMT-sMF	4.76	-16.65 to 26.17	>1.00	0.171
	IPC vs. ESWT	6.60	-9.89 to 23.09	>1.00	0.194
	PR vs. ESWT	10.75	-7.53 to 29.02	0.61	0.354
	PR vs. IPC	4.15	-11.42 to 19.72	>1.00	0.190
24 hours	ESWT vs. PBMT-sMF	31.71	8.84 to 54.57	0.00	2.020
	IPC vs. PBMT-sMF	34.76	17.13 to 52.39	<0.00	1.995
	PR vs. PBMT-sMF	44.74	24.30 to 65.18	<0.00	1.779
	IPC vs. ESWT	3.05	-17.78 to 23.89	>1.00	0.096
	PR vs. ESWT	13.04	-9.98 to 36.05	0.68	0.337
	PR vs. IPC	9.98	-7.88 to 27.85	0.70	0.502
48 hours	ESWT vs. PBMT-sMF	64.14	47.42 to 80.86	<0.00	3.173
	IPC vs. PBMT-sMF	70.09	50.96 to 89.23	<0.00	4.240
	PR vs. PBMT-sMF	78.69	60.28 to 97.09	<0.00	3.451
	IPC vs. ESWT	5.95	-15.48 to 27.38	>1.00	0.019
	PR vs. ESWT	14.54	-6.30 to 35.39	0.33	0.344
	PR vs. IPC	8.59	-13.96 to 31.15	>1.00	0.348

Values are presented as mean differences (MD) with 95% confidence intervals (CI). Effect sizes were calculated using Cohen's d. Time points included baseline. 1 hour. 24 hours. and 48 hours. PR = passive recovery; IPC = intermittent pneumatic compression; ESWT = extracorporeal shockwave therapy; PBMT-sMF = photobiomodulation therapy combined with static magnetic field.

Table 6. Between-intervention comparisons of TBARS values across time points.

Time point	Comparisons	Mean difference	95% CI	p- value	Effect Size
Baseline	ESWT vs. PBMT-sMF	-0.01	-0.37 to 0.35	>1.00	0.053
	IPC vs. PBMT-sMF	-0.08	-0.42 to 0.27	>1.00	0.224
	PR vs. PBMT-sMF	-0.25	-0.61 to 0.11	0.29	0.579
	IPC vs. ESWT	-0.06	-0.30 to 0.17	>1.00	0.204
	PR vs. ESWT	-0.24	-0.50 to 0.02	0.07	0.822
	PR vs. IPC	-0.18	-0.41 to 0.05	0.20	0.763
1 hour	ESWT vs. PBMT-sMF	-0.05	-0.54 to 0.44	>1.00	0.084
	IPC vs. PBMT-sMF	-0.03	-0.47 to 0.42	>1.00	0.074
	PR vs. PBMT-sMF	-0.16	-0.60 to 0.24	>1.00	0.331
	IPC vs. ESWT	0.02	-0.45 to 0.49	>1.00	0.028
	PR vs. ESWT	-0.16	-0.55 to 0.32	>1.00	0.241
	PR vs. IPC	-0.13	-0.51 to 0.24	>1.00	0.250
24 hours	ESWT vs. PBMT-sMF	1.24	0.72 to 1.76	<0.00	2.614
	IPC vs. PBMT-sMF	1.08	0.73 to 1.44	<0.00	2.737
	PR vs. PBMT-sMF	1.08	0.65 to 1.51	<0.00	2.168
	IPC vs. ESWT	-0.15	-0.71 to 0.40	>1.00	0.359
	PR vs. ESWT	-0.16	-0.75 to 0.44	>1.00	0.208
	PR vs. IPC	-0.00	-0.48 to 0.47	>1.00	0.000
48 hours	ESWT vs. PBMT-sMF	2.35	1.92 to 2.78	<0.00	4.434
	IPC vs. PBMT-sMF	2.20	1.84 to 2.56	<0.00	4.931
	PR vs. PBMT-sMF	2.09	1.77 to 2.42	<0.00	5.195
	IPC vs. ESWT	-0.15	-0.62 to 0.33	>1.00	0.344
	PR vs. ESWT	-0.26	-0.71 to 0.20	0.70	0.703
	PR vs. IPC	-0.11	-0.51 to 0.29	>1.00	0.249

Values are presented as mean differences (MD) with 95% confidence intervals (CI). Effect sizes were calculated using Cohen’s d. Time points included baseline. 1 hour. 24 hours. and 48 hours. PR = passive recovery; IPC = intermittent pneumatic compression; ESWT = extracorporeal shockwave therapy; PBMT-sMF = photobiomodulation therapy combined with static magnetic field.

Table 7. Between-intervention comparisons of changes in carbonylated protein across time points.

Time point	Comparisons	Mean difference	95% CI	p- value	Effect Size
Baseline	ESWT vs. PBMT-sMF	0.00	-	-	-
	IPC vs. PBMT-sMF	0.00	-	-	-
	PR vs. PBMT-sMF	0.00	-	-	-
	IPC vs. ESWT	0.00	-	-	-
	PR vs. ESWT	0.00	-	-	-
	PR vs. IPC	0.00	-	-	-
1 hour	ESWT vs. PBMT-sMF	7.63	-8.30 to 23.57	>1.00	0.392
	IPC vs. PBMT-sMF	2.92	-12.64 to 18.48	>1.00	0.162
	PR vs. PBMT-sMF	9.79	-9.39 to 28.97	0.89	0.708
	IPC vs. ESWT	-4.72	-22.09 to 12.66	>1.00	0.264
	PR vs. ESWT	2.16	-18.32 to 22.63	>1.00	0.088
	PR vs. IPC	6.87	-13.37 to 27.11	>1.00	0.351
24 hours	ESWT vs. PBMT-sMF	20.26	-2.95 to 43.46	0.11	0.860
	IPC vs. PBMT-sMF	19.19	-2.29 to 40.68	0.01	0.635
	PR vs. PBMT-sMF	23.74	4.09 to 43.39	0.01	1.191
	IPC vs. ESWT	-1.06	-26.14 to 24.02	>1.00	0.040
	PR vs. ESWT	3.49	-20.24 to 27.21	>1.00	0.148
	PR vs. IPC	4.55	-17.53 to 26.63	>1.00	0.228
48 hours	ESWT vs. PBMT-sMF	28.49	8.53 to 48.45	0.00	1.312
	IPC vs. PBMT-sMF	17.43	-3.48 to 38.35	0.15	0.790
	PR vs. PBMT-sMF	27.18	5.37 to 49.00	0.01	1.106
	IPC vs. ESWT	-11.06	-31.10 to 8.97	0.74	0.487
	PR vs. ESWT	-1.31	-22.31 to 19.69	>1.00	0.058
	PR vs. IPC	9.75	-12.13 to 31.63	>1.00	0.428

Values are presented as mean differences (MD) with 95% confidence intervals (CI). Effect sizes were calculated using Cohen’s d. Time points included baseline. 1 hour. 24 hours. and 48 hours. PR = passive recovery; IPC = intermittent pneumatic compression; ESWT = extracorporeal shockwave therapy; PBMT-sMF = photobiomodulation therapy combined with static magnetic field.

Table 8. Between-intervention comparisons of carbonylated protein values across time points.

Time point	Comparisons	Mean difference	95% CI	p- value	Effect Size
Baseline	ESWT vs. PBMT-sMF	-0.06	-0.56 to 0.43	>1.00	0.124
	IPC vs. PBMT-sMF	-0.03	-0.43 to 0.38	>1.00	0.061
	PR vs. PBMT-sMF	-0.27	-0.75 to 0.20	0.66	0.648
	IPC vs. ESWT	0.04	-0.46 to 0.53	>1.00	0.063
	PR vs. ESWT	-0.21	-0.75 to 0.33	>1.00	0.373
	PR vs. IPC	-0.25	-0.72 to 0.23	0.87	0.591
1 hour	ESWT vs. PBMT-sMF	0.29	-0.17 to 0.75	0.49	0.404
	IPC vs. PBMT-sMF	0.12	-0.46 to 0.69	>1.00	0.188
	PR vs. PBMT-sMF	0.14	-0.41 to 0.68	>1.00	0.303
	IPC vs. ESWT	-0.17	-0.73 to 0.38	>1.00	0.220
	PR vs. ESWT	-0.15	-0.68 to 0.37	>1.00	0.229
	PR vs. IPC	0.02	-0.60 to 0.64	>1.00	0.030
24 hours	ESWT vs. PBMT-sMF	0.92	0.25 to 1.59	0.00	1.212
	IPC vs. PBMT-sMF	0.95	0.27 to 1.64	0.00	0.952
	PR vs. PBMT-sMF	0.89	0.08 to 1.71	0.03	0.962
	IPC vs. ESWT	0.03	-0.71 to 0.76	>1.00	0.033
	PR vs. ESWT	-0.03	-0.89 to 0.82	>1.00	0.028
	PR vs. IPC	-0.06	-0.92 to 0.80	>1.00	0.067
48 hours	ESWT vs. PBMT-sMF	1.38	0.63 to 2.14	0.00	1.506
	IPC vs. PBMT-sMF	0.88	0.06 to 1.70	0.03	1.088
	PR vs. PBMT-sMF	1.01	0.29 to 1.74	0.00	1.184
	IPC vs. ESWT	-0.51	-1.33 to 0.31	0.51	0.454
	PR vs. ESWT	-0.37	-1.10 to 0.35	0.90	0.411
	PR vs. IPC	0.13	-0.66 to 0.93	>1.00	0.139

Values are presented as mean differences (MD) with 95% confidence intervals (CI). Effect sizes were calculated using Cohen's d. Time points included baseline. 1 hour. 24 hours. and 48 hours. PR = passive recovery; IPC = intermittent pneumatic compression; ESWT = extracorporeal shockwave therapy; PBMT-sMF = photobiomodulation therapy combined with static magnetic field.

Table 9. Between-intervention comparisons of changes in CAT across time points.

Time point	Comparisons	Mean difference	95% CI	p- value	Effect Size
Baseline	ESWT vs. PBMT-sMF	0.00	-	-	-
	IPC vs. PBMT-sMF	0.00	-	-	-
	PR vs. PBMT-sMF	0.00	-	-	-
	IPC vs. ESWT	0.00	-	-	-
	PR vs. ESWT	0.00	-	-	-
	PR vs. IPC	0.00	-	-	-
1 hour	ESWT vs. PBMT-sMF	-7.13	-26.41 to 12.15	>1.00	0.291
	IPC vs. PBMT-sMF	-11.00	-31.31 to 9.31	0.77	0.407
	PR vs. PBMT-sMF	-15.92	-35.24 to 3.41	0.155	0.959
	IPC vs. ESWT	-3.87	-25.84 to 18.09	>1.00	0.174
	PR vs. ESWT	-8.79	-29.90 to 12.33	>1.00	0.332
	PR vs. IPC	-4.91	-26.92 to 17.09	>1.00	0.158
24 hours	ESWT vs. PBMT-sMF	-40.90	-57.14 to -24.66	<0.00	2.152
	IPC vs. PBMT-sMF	-46.20	-61.89 to -30.52	<0.00	3.358
	PR vs. PBMT-sMF	-41.13	-60.09 to -22.16	<0.00	2.250
	IPC vs. ESWT	-5.30	-18.50 to 7.90	>1.00	0.378
	PR vs. ESWT	-0.22	-17.53 to 17.08	>1.00	0.010
	PR vs. IPC	5.08	-11.74 to 21.89	>1.00	0.271
48 hours	ESWT vs. PBMT-sMF	-40.17	-57.25 to -23.10	<0.00	1.890
	IPC vs. PBMT-sMF	-46.98	-65.10 to -28.86	<0.00	2.300
	PR vs. PBMT-sMF	-45.81	-61.91 to -29.71	<0.00	2.607
	IPC vs. ESWT	-6.81	-22.95 to 9.33	>1.00	0.287
	PR vs. ESWT	-5.63	-19.16 to 7.89	>1.00	0.354
	PR vs. IPC	1.17	-13.87 to 16.22	>1.00	0.056

Values are presented as mean differences (MD) with 95% confidence intervals (CI). Effect sizes were calculated using Cohen's d. Time points included baseline. 1 hour. 24 hours. and 48 hours. PR = passive recovery; IPC = intermittent pneumatic compression; ESWT = extracorporeal shockwave therapy; PBMT-sMF = photobiomodulation therapy combined with static magnetic field.

Table 10. Between-intervention comparisons of CAT values across time points.

Time point	Comparisons	Mean difference	95% CI	p- value	Effect Size
Baseline	ESWT vs. PBMT-sMF	0.02	-0.43 to 0.46	>1.00	0.016
	IPC vs. PBMT-sMF	0.21	-0.28 to 0.70	>1.00	0.323
	PR vs. PBMT-sMF	0.24	-0.36 to 0.85	>1.00	0.461
	IPC vs. ESWT	0.20	-0.23 to 0.69	>1.00	0.312
	PR vs. ESWT	0.23	-0.38 to 0.84	>1.00	0.327
	PR vs. IPC	0.03	-0.60 to 0.67	>1.00	0.033
1 hour	ESWT vs. PBMT-sMF	-0.29	-0.81 to 0.23	0.70	0.554
	IPC vs. PBMT-sMF	-0.27	-0.76 to 0.22	0.73	0.480
	PR vs. PBMT-sMF	-0.48	-0.92 to -0.03	0.03	0.983
	IPC vs. ESWT	0.02	-0.52 to 0.57	>1.00	0.031
	PR vs. ESWT	-0.18	-0.70 to 0.33	>1.00	0.333
	PR vs. IPC	-0.21	-0.68 to 0.27	>1.00	0.354
24 hours	ESWT vs. PBMT-sMF	-1.70	-2.20 to -1.20	<0.00	4.117
	IPC vs. PBMT-sMF	-1.79	-2.21 to -1.37	<0.00	4.324
	PR vs. PBMT-sMF	-1.58	-2.03 to -1.14	<0.00	3.461
	IPC vs. ESWT	-0.09	-0.56 to 0.38	>1.00	0.134
	PR vs. ESWT	0.12	-0.37 to 0.61	>1.00	0.178
	PR vs. IPC	0.20	-0.21 to 0.61	0.98	0.498
48 hours	ESWT vs. PBMT-sMF	-1.67	-2.19 to -1.16	<0.00	2.598
	IPC vs. PBMT-sMF	-1.85	-2.34 to -1.36	<0.00	3.493
	PR vs. PBMT-sMF	-1.77	-2.16 to -1.37	<0.00	2.971
	IPC vs. ESWT	-0.17	-0.71 to 0.37	>1.00	0.231
	PR vs. ESWT	-0.10	-0.56 to 0.37	>1.00	0.185
	PR vs. IPC	0.08	-0.35 to 0.51	>1.00	0.144

Values are presented as mean differences (MD) with 95% confidence intervals (CI). Effect sizes were calculated using Cohen's d. Time points included baseline. 1 hour. 24 hours. and 48 hours. PR = passive recovery; IPC = intermittent pneumatic compression; ESWT = extracorporeal shockwave therapy; PBMT-sMF = photobiomodulation therapy combined with static magnetic field.

Table 11. Between-intervention comparisons of changes in SOD across time points.

Time point	Comparisons	Mean difference	95% CI	p- value	Effect Size
Baseline	ESWT vs. PBMT-sMF	0.00	-	-	-
	IPC vs. PBMT-sMF	0.00	-	-	-
	PR vs. PBMT-sMF	0.00	-	-	-
	IPC vs. ESWT	0.00	-	-	-
	PR vs. ESWT	0.00	-	-	-
	PR vs. IPC	0.00	-	-	-
1 hour	ESWT vs. PBMT-sMF	-8.44	-25.10 to 8.22	0.94	0.386
	IPC vs. PBMT-sMF	-13.81	-28.94 to 1.32	0.09	0.777
	PR vs. PBMT-sMF	-18.04	-37.04 to 0.96	0.07	0.943
	IPC vs. ESWT	-5.37	-19.51 to 8.76	>1.00	0.359
	PR vs. ESWT	-9.60	-27.96 to 8.76	0.85	0.474
	PR vs. IPC	-4.23	-21.33 to 12.88	>1.00	0.258
24 hours	ESWT vs. PBMT-sMF	-29.79	-46.81 to -12.78	0.00	1.612
	IPC vs. PBMT-sMF	-38.60	-57.67 to -19.53	<0.00	1.509
	PR vs. PBMT-sMF	-33.23	-49.35 to -17.10	<0.00	1.436
	IPC vs. ESWT	-8.81	-26.17 to 8.56	0.92	0.402
	PR vs. ESWT	-3.43	-17.12 to 10.25	>1.00	0.182
	PR vs. IPC	5.37	-11.14 to 21.89	>1.00	0.327
48 hours	ESWT vs. PBMT-sMF	-39.11	-59.95 to -18.27	0.00	1.792
	IPC vs. PBMT-sMF	-45.71	-65.93 to -25.48	<0.00	2.010
	PR vs. PBMT-sMF	-45.96	-66.40 to -25.52	<0.00	1.966
	IPC vs. ESWT	-6.60	-23.71 to 10.52	>1.00	0.301
	PR vs. ESWT	-6.85	-24.26 to 10.56	>1.00	0.326
	PR vs. IPC	-0.26	-16.80 to 16.28	>1.00	0.013

Values are presented as mean differences (MD) with 95% confidence intervals (CI). Effect sizes were calculated using Cohen's d. Time points included baseline. 1 hour. 24 hours. and 48 hours. PR = passive recovery; IPC = intermittent pneumatic compression; ESWT = extracorporeal shockwave therapy; PBMT-sMF = photobiomodulation therapy combined with static magnetic field.

Table 12. Between-intervention comparisons of SOD values across time points.

Time point	Comparisons	Mean difference	95% CI	p- value	Effect Size
Baseline	ESWT vs. PBMT-sMF	-0.03	-0.47 to 0.40	>1.00	0.058
	IPC vs. PBMT-sMF	0.05	-0.41 to 0.51	>1.00	0.081
	PR vs. PBMT-sMF	0.11	-0.34 to 0.57	>1.00	0.225
	IPC vs. ESWT	0.08	-0.30 to 0.47	>1.00	0.144
	PR vs. ESWT	0.15	-0.23 to 0.52	>1.00	0.288
	PR vs. IPC	0.06	-0.34 to 0.46	>1.00	0.116
1 hour	ESWT vs. PBMT-sMF	-0.31	-0.76 to 0.13	0.33	0.541
	IPC vs. PBMT-sMF	-0.39	-0.89 to 0.10	0.18	0.636
	PR vs. PBMT-sMF	-0.51	-1.01 to -0.01	0.04	0.954
	IPC vs. ESWT	-0.08	-0.53 to 0.37	>1.00	0.145
	PR vs. ESWT	-0.20	-0.65 to 0.26	>1.00	0.426
	PR vs. IPC	-0.12	-0.62 to 0.38	>1.00	0.239
24 hours	ESWT vs. PBMT-sMF	-1.01	-1.49 to -0.54	<0.00	2.080
	IPC vs. PBMT-sMF	-1.26	-1.75 to -0.76	<0.00	1.876
	PR vs. PBMT-sMF	-0.99	-1.48 to -0.50	<0.00	1.526
	IPC vs. ESWT	-0.24	-0.77 to 0.29	>1.00	0.368
	PR vs. ESWT	0.03	-0.50 to 0.55	>1.00	0.043
	PR vs. IPC	0.27	-0.27 to 0.81	0.98	0.406
48 hours	ESWT vs. PBMT-sMF	-1.33	-1.82 to -0.83	<0.00	2.226
	IPC vs. PBMT-sMF	-1.48	-1.94 to -1.01	<0.00	2.449
	PR vs. PBMT-sMF	-1.44	-1.91 to -0.98	<0.00	2.956
	IPC vs. ESWT	-0.15	-0.64 to 0.34	>1.00	0.296
	PR vs. ESWT	-0.11	-0.60 to 0.37	>1.00	0.230
	PR vs. IPC	0.04	-0.42 to 0.49	>1.00	0.054

Values are presented as mean differences (MD) with 95% confidence intervals (CI). Effect sizes were calculated using Cohen's d. Time points included baseline. 1 hour. 24 hours. and 48 hours. PR = passive recovery; IPC = intermittent pneumatic compression; ESWT = extracorporeal shockwave therapy; PBMT-sMF = photobiomodulation therapy combined with static magnetic field.

Table 13. Between-intervention comparisons of respiratory RPE across time points.

Time point	Comparisons	Mean difference	95% CI	p- value	Effect Size
Baseline	ESWT vs. PBMT-sMF	-3.04	-9.39 to 3.31	0.92	0.432
	IPC vs. PBMT-sMF	1.42	-8.50 to 11.33	>1.00	0.120
	PR vs. PBMT-sMF	-2.83	-9.21 to 3.54	>1.00	0.400
	IPC vs. ESWT	4.46	-4.37 to 13.28	0.80	0.480
	PR vs. ESWT	0.21	-1.19 to 1.60	>1.00	0.123
	PR vs. IPC	-4.25	-13.09 to 4.59	0.92	0.434
After WOD	ESWT vs. PBMT-sMF	-0.25	-29.31 to 28.81	>1.00	0.018
	IPC vs. PBMT-sMF	-0.17	-29.26 to 28.93	>1.00	0.010
	PR vs. PBMT-sMF	5.83	-19.96 to 31.63	>1.00	0.411
	IPC vs. ESWT	0.08	-27.17 to 27.33	>1.00	0.005
	PR vs. ESWT	6.08	-17.36 to 29.52	>1.00	0.380
	PR vs. IPC	6.00	-17.49 to 29.49	>1.00	0.555
After treatment	ESWT vs. PBMT-sMF	-2.17	-9.75 to 5.42	>1.00	0.258
	IPC vs. PBMT-sMF	0.17	-9.60 to 9.94	>1.00	0.019
	PR vs. PBMT-sMF	5.17	-10.61 to 20.94	>1.00	0.390
	IPC vs. ESWT	2.33	-6.39 to 11.06	>1.00	0.226
	PR vs. ESWT	7.33	-8.06 to 22.72	0.97	0.489
	PR vs. IPC	5.00	-11.11 to 21.11	>1.00	0.361
1 hour	ESWT vs. PBMT-sMF	-0.17	-4.79 to 4.46	>1.00	0.058
	IPC vs. PBMT-sMF	2.83	-5.22 to 10.88	>1.00	0.302
	PR vs. PBMT-sMF	7.17	-8.61 to 22.94	>1.00	0.520
	IPC vs. ESWT	3.00	-4.81 to 10.81	>1.00	0.341
	PR vs. ESWT	7.33	-8.38 to 23.04	1.00	0.484
	PR vs. IPC	4.33	-12.00 to 20.67	>1.00	0.285
24 hours	ESWT vs. PBMT-sMF	0.58	-2.72 to23.88	>1.00	0.287
	IPC vs. PBMT-sMF	0.96	-1.94 to 3.86	>1.00	0.273
	PR vs. PBMT-sMF	0.42	-2.38 to 3.21	>1.00	0.123
	IPC vs. ESWT	0.37	-3.47 to 4.22	>1.00	0.078
	PR vs. ESWT	-0.1667	-3.95 to 3.62	>1.00	0.036

	PR vs. IPC	-0.5417	-4.03 to 2.95	>1.00	0.140
48 hours	ESWT vs. PBMT-sMF	0.8333	-1.84 to 3.51	>1.00	0.287
	IPC vs. PBMT-sMF	0.2500	-0.55 to 1.05	>1.00	0.287
	PR vs. PBMT-sMF	0.5000	-1.10 to 2.10	>1.00	0.289
	IPC vs. ESWT	-0.5833	-3.29 to 2.12	>1.00	0.188
	PR vs. ESWT	-0.3333	-3.21 to 2.55	>1.00	0.094
	PR vs. IPC	0.2500	-1.43 to 1.93	>1.00	0.125

Values are presented as mean differences (MD) with 95% confidence intervals (CI). Effect sizes were calculated using Cohen's d. Time points included baseline. 1 hour. 24 hours. and 48 hours. PR = passive recovery; IPC = intermittent pneumatic compression; ESWT = extracorporeal shockwave therapy; PBMT-sMF = photobiomodulation therapy combined with static magnetic field.

Table 14. Between-intervention comparisons of muscle RPE across time points.

Time point	Comparisons	Mean difference	95% CI	p- value	Effect Size
Baseline	ESWT vs. PBMT-sMF	-5.83	-27.22 to 15.56	>1.00	0.267
	IPC vs. PBMT-sMF	-7.17	-24.69 to 10.36	>1.00	0.408
	PR vs. PBMT-sMF	-13.63	-29.31 to 2.06	0.11	0.951
	IPC vs. ESWT	-1.33	-21.37 to 18.71	>1.00	0.100
	PR vs. ESWT	-7.79	-26.44 to 10.86	>1.00	0.502
	PR vs. IPC	-6.46	-19.53 to 6.62	0.96	0.701
After WOD	ESWT vs. PBMT-sMF	-2.25	-20.09 to 15.59	>1.00	0.212
	IPC vs. PBMT-sMF	-3.50	-23.43 to 16.43	>1.00	0.338
	PR vs. PBMT-sMF	-12.25	-36.36 to 11.86	0.89	0.504
	IPC vs. ESWT	-1.25	-22.25 to 19.75	>1.00	0.100
	PR vs. ESWT	-10.00	-34.87 to 14.87	>1.00	0.552
	PR vs. IPC	-8.75	-34.84 to 17.34	>1.00	0.437
After treatment	ESWT vs. PBMT-sMF	-2.17	-23.66 to 19.33	>1.00	0.084
	IPC vs. PBMT-sMF	-5.25	-24.38 to 13.88	>1.00	0.393
	PR vs. PBMT-sMF	-2.33	-22.39 to 17.73	>1.00	0.092
	IPC vs. ESWT	-3.08	-27.28 to 21.11	>1.00	0.139
	PR vs. ESWT	-0.17	-24.98 to 24.65	>1.00	0.005
	PR vs. IPC	2.91	-20.14 to 25.98	>1.00	0.094
1 hour	ESWT vs. PBMT-sMF	-1.75	-27.93 to 24.43	>1.00	0.056
	IPC vs. PBMT-sMF	-1.83	-22.53 to 18.86	>1.00	0.164
	PR vs. PBMT-sMF	0.33	-23.28 to 23.95	>1.00	0.010
	IPC vs. ESWT	-0.08	-26.83 to 26.66	>1.00	0.003
	PR vs. ESWT	2.08	-26.62 to 30.79	>1.00	0.058
	PR vs. IPC	2.17	-22.13 to 26.47	>1.00	0.064
24 hours	ESWT vs. PBMT-sMF	-6.08	-24.36 to 12.19	>1.00	0.275
	IPC vs. PBMT-sMF	-1.54	-19.69 to 16.61	>1.00	0.115
	PR vs. PBMT-sMF	1.17	-21.39 to 23.72	>1.00	0.044
	IPC vs. ESWT	4.54	-13.41 to 22.50	>1.00	0.245
	PR vs. ESWT	7.25	-15.18 to 29.68	>1.00	0.305

	PR vs. IPC	2.71	-19.63 to 25.05	>1.00	0.098
48 hours	ESWT vs. PBMT-sMF	-1.75	-15.73 to 12.23	>1.00	0.096
	IPC vs. PBMT-sMF	-3.46	-19.43 to 12.51	>1.00	0.333
	PR vs. PBMT-sMF	0.58	-18.10 to 19.27	>1.00	0.030
	IPC vs. ESWT	-1.71	-16.78 to 13.36	>1.00	0.102
	PR vs. ESWT	2.33	-15.69 to 20.36	>1.00	0.115
	PR vs. IPC	4.04	-15.32 to 23.40	>1.00	0.176

Values are presented as mean differences (MD) with 95% confidence intervals (CI). Effect sizes were calculated using Cohen's d. Time points included baseline. 1 hour. 24 hours. and 48 hours. PR = passive recovery; IPC = intermittent pneumatic compression; ESWT = extracorporeal shockwave therapy; PBMT-sMF = photobiomodulation therapy combined with static magnetic field.
