# Supplementary material for: A randomised crossover trial comparing photobiomodulation therapy with other recovery strategies in CrossFit athletes
Source: PLoS One. 2026 May 22;21(5):e0349880. doi: 10.1371/journal.pone.0349880 (PMC13196929; doi:10.1371/journal.pone.0349880)
Supplement: S2 Table — (PDF) [file pone.0349880.s003.pdf]

WOD - Treatment (time in seconds)

ID	PBMT-sMF	Shock Wave	Passive Recovery	Pneumatic Compression
1	569	510	467	559
2	516	580	561	554
3	506	779	619	489
4	587	611	541	560
5	386	330	348	354
6	447	445	420	460
7	588	599	540	595
8	600	676	597	539
9	445	419	419	395
10	476	483	536	543
11	608	673	645	625
12	500	461	454	440

WOD - Weeks (time in seconds)

ID	Week 1	Week 2	Week 3	Week 4
1	559	569	510	467
2	580	561	554	516
3	779	619	489	506
4	587	611	541	560
5	348	354	386	330
6	460	447	445	420
7	599	540	595	588
8	676	597	539	600
9	419	395	445	419
10	536	543	476	483
11	645	625	608	673
12	500	461	454	440

CMJ

	PBMT-sMF				Shock Wave				Passive Recovery				Pneumatic Compression			
**ID**	Baseline	1h	24h	48h	Baseline	1h	24h	48h	Baseline	1h	24h	48h	Baseline	1h	24h	48h
1	42.87	40.48	39.9	40.48	39.9	40.48	40.48	38.74	42.26	41.67	37.6	37.04	42.2	41.01	39.9	41.67
2	44.7	42.87	39.9	42.26	42.26	36.48	41.07	38.84	45.94	45.94	43.41	38.74	41.67	44.08	38.74	38.74
3	45.94	46.57	47.84	48.48	47.2	41.67	43.47	44.7	44.7	40.48	41.67	44.08	44.7	39.32	41.67	41.07
4	39.9	35.92	39.9	38.74	40.43	38.74	35.87	38.17	38.74	37.04	39.32	35.37	42.26	42.87	42.26	41.07
5	37.04	37.04	33.74	33.74	37.04	40.48	40.48	37.6	35.92	37.6	33.74	33.21	34.28	36.92	32.16	32.16
6	42.26	41.67	44.7	41.67	45.32	41.67	42.2	43.47	46.51	44.08	44.08	37.04	44.7	44.08	45.32	42.87
7	41.01	43.47	45.32	44.7	44.08	41.67	41.07	39.26	38.17	35.92	35.37	34.28	39.32	36.48	38.17	41.07
8	40.48	43.47	40.48	41.67	44.7	38.74	41.07	37.6	45.32	39.9	40.48	39.9	39.9	40.48	40.48	39.32
9	45.94	40.43	46.57	45.32	46.57	44.08	48.48	47.2	54.43	47.84	50.42	49.06	53.08	41.67	47.84	41.07
10	38.74	41.01	41.67	42.87	39.9	38.17	37.6	37.04	37.6	38.74	35.37	34.83	39.9	37.04	40.48	39.9
11	41.01	45.88	38.74	42.26	42.87	41.67	41.67	42.87	43.47	40.48	41.07	39.32	44.7	45.94	42.81	41.07
12	39.32	41.67	38.74	39.32	41.07	40.48	39.9	40.48	44.08	39.9	42.26	43.47	41.07	39.26	39.9	42.87

LDH

	PBMT-sMF				Shock Wave				Passive recovery				Pneumatic compression			
**ID**	Baseline	1h	24h	48h	Baseline	1h	24h	48h	Baseline	1h	24h	48h	Baseline	1h	24h	48h
1	162.16	148.07	237.88	364.03	153.91	180.09	307.00	490.11	192.50	197.87	193.15	212.93	135.78	228.42	334.33	393.93
2	199.61	238.65	254.19	390.35	197.21	221.41	318.40	457.17	141.05	323.27	352.56	485.09	269.23	297.27	373.94	506.73
3	215.18	297.06	266.76	379.47	216.06	237.96	323.01	462.79	157.57	197.46	201.80	431.29	214.34	301.59	319.14	433.71
4	176.40	224.90	219.69	383.93	289.65	303.80	340.32	553.08	167.67	188.70	315.34	528.56	186.07	258.55	298.71	539.01
5	138.44	222.84	199.44	358.61	137.51	281.32	302.28	438.92	236.72	259.26	334.72	519.97	130.64	190.95	306.33	396.85
6	198.14	194.38	258.36	378.87	172.96	192.22	328.79	514.69	237.66	299.52	390.17	486.35	202.30	223.83	324.90	482.85
7	266.58	201.40	268.85	617.01	150.61	235.47	270.21	417.31	184.66	310.29	350.09	530.34	155.78	217.99	295.00	514.61
8	218.98	266.11	210.02	422.78	106.66	188.47	202.33	401.74	160.38	180.73	306.28	512.68	148.12	243.52	308.88	377.94
9	198.92	222.10	345.78	420.58	246.12	295.31	283.15	525.21	132.11	235.67	278.37	410.47	115.96	200.60	286.44	365.26
10	232.92	194.71	248.71	407.06	98.59	165.90	230.63	278.78	183.64	251.23	297.49	510.22	149.30	282.55	165.96	435.77
11	173.55	175.22	253.90	425.75	187.50	252.22	318.19	502.76	193.33	222.07	326.26	533.58	140.05	244.95	278.84	506.33
12	192.61	294.30	246.15	406.22	196.34	290.12	385.05	435.34	176.75	236.35	326.62	468.35	161.87	232.69	345.28	498.61

TBARS

	Passive recovery				Shock wave				Pneumatic compression				PBMT-sMF			
**ID**	Baseline	1h	24h	48h	Baseline	1h	24h	48h	Baseline	1h	24h	48h	Baseline	1h	24h	48h
1	3.00	4.45	5.00	6.41	3.18	4.56	6.51	6.92	3.04	4.14	5.55	6.93	3.11	4.45	4.80	4.63
2	3.30	4.37	5.17	6.90	3.26	5.31	5.57	7.94	3.33	4.87	5.18	7.37	2.99	4.41	4.51	4.36
3	2.95	4.78	4.94	6.59	3.36	4.87	5.62	7.07	3.14	3.64	5.44	7.19	3.94	3.83	4.33	4.86
4	3.18	4.40	6.20	6.28	3.42	3.87	6.93	6.45	3.55	4.48	6.38	6.48	3.32	4.47	4.77	4.46
5	3.30	4.42	5.37	6.43	3.66	4.47	5.91	7.15	3.42	4.41	5.43	6.40	3.66	4.97	4.19	4.79
6	2.62	4.44	5.67	6.54	3.47	4.13	5.42	6.47	3.27	4.41	5.75	6.77	3.44	5.01	4.19	4.51
7	3.07	3.93	5.38	6.99	3.40	4.51	6.03	7.06	3.08	4.91	5.88	7.24	2.91	4.78	4.40	4.53
8	3.41	4.48	5.58	6.72	3.49	4.86	5.13	6.46	3.32	4.79	5.63	6.50	3.67	4.54	4.33	5.03
9	3.34	4.08	6.43	6.72	3.16	4.13	5.37	6.69	3.30	4.27	5.38	7.04	2.91	4.38	4.39	4.40
10	3.04	4.22	5.84	7.00	3.03	3.84	5.20	7.04	3.48	4.64	5.27	6.69	3.84	3.82	4.38	4.76
11	3.00	4.55	5.50	7.36	3.08	4.27	5.32	7.42	3.42	4.74	5.60	6.39	3.20	4.93	4.44	4.43
12	3.13	3.92	5.48	6.94	3.74	4.60	5.42	7.28	3.13	4.34	5.09	7.20	3.40	4.38	4.83	4.98

Carbonylated protein

	Passive recovery				Shock wave				Pneumatic compression				PBMT-sMF			
**ID**	Baseline	1h	24h	48h	Baseline	1h	24h	48h	Baseline	1h	24h	48h	Baseline	1h	24h	48h
1	4.38	5.71	6.69	6.57	5.24	6.30	5.79	7.14	5.19	4.54	5.58	6.67	4.54	5.55	5.81	6.12
2	5.04	5.10	6.29	6.31	5.13	5.58	7.17	5.77	5.23	5.76	5.84	6.56	5.44	5.68	5.04	5.15
3	5.84	5.78	7.72	5.45	5.69	6.73	5.80	7.31	5.53	5.68	6.32	6.41	5.52	4.89	5.43	6.19
4	5.00	5.89	6.03	6.76	5.23	5.53	6.23	7.16	5.21	6.02	7.43	5.76	5.70	5.75	4.37	4.64
5	4.84	5.40	6.11	6.94	4.28	6.53	6.74	7.56	5.48	6.33	5.66	6.86	5.05	5.29	6.12	5.47
6	4.94	5.52	4.80	6.54	5.52	5.95	6.02	7.53	5.12	5.15	6.14	6.50	5.19	6.17	5.52	4.60
7	5.13	5.79	5.59	7.49	5.43	6.16	6.07	7.67	5.30	5.77	6.16	5.28	5.24	5.44	5.21	6.09
8	4.13	6.55	6.57	7.08	4.72	6.06	7.22	6.40	4.25	6.04	7.30	6.05	5.31	6.11	5.34	5.06
9	4.66	6.09	6.29	6.74	5.39	5.65	5.68	6.43	5.26	6.52	6.60	7.45	5.09	6.15	6.00	5.81
10	5.05	6.01	6.64	5.86	5.44	5.96	5.71	7.80	5.05	6.24	6.91	6.60	5.44	6.08	4.82	5.53
11	5.26	5.62	7.55	7.34	4.21	5.96	6.65	6.38	4.80	6.22	6.87	7.93	4.88	5.54	5.75	6.59
12	4.51	6.97	5.64	6.28	5.01	5.87	7.22	6.71	5.31	5.93	5.82	5.70	4.66	6.15	5.80	5.96

CAT

	Passive recovery				Shock wave				Pneumatic compression				PBMT-sMF			
**ID**	Baseline	1h	24h	48h	Baseline	1h	24h	48h	Baseline	1h	24h	48h	Baseline	1h	24h	48h
1	3.83	4.77	4.20	2.98	4.09	4.64	3.46	3.87	4.59	5.46	4.00	2.85	3.93	4.83	5.38	5.36
2	4.66	4.39	3.64	3.28	4.29	4.05	3.32	3.70	4.23	4.38	3.17	3.34	4.82	4.00	5.08	4.93
3	5.09	4.06	3.05	3.84	4.35	4.86	3.71	4.13	4.73	4.46	3.31	2.87	4.29	5.02	4.60	4.09
4	4.09	5.33	3.92	3.51	3.77	4.67	2.85	3.08	4.90	4.21	3.55	2.76	3.87	5.12	5.13	5.03
5	3.51	4.37	3.50	3.05	4.36	3.69	3.50	3.18	4.62	4.68	3.79	3.72	4.07	5.10	5.70	5.26
6	4.97	4.45	3.33	3.25	4.57	4.79	4.19	3.80	4.05	4.87	3.81	3.35	3.87	4.94	5.58	5.66
7	5.33	4.42	4.11	3.50	3.64	5.37	4.19	2.73	3.65	5.62	3.31	3.63	4.93	4.95	5.74	5.15
8	4.87	5.03	3.65	3.33	3.94	5.17	3.40	3.94	4.82	4.83	2.97	2.53	4.68	5.46	4.92	5.25
9	4.93	4.47	3.86	3.37	4.50	5.22	4.07	2.85	4.90	4.33	3.33	3.73	4.32	4.70	5.60	4.81
10	4.85	4.08	3.37	2.99	4.93	4.39	4.17	3.45	3.77	4.65	3.41	3.56	3.95	4.91	5.52	5.40
11	4.15	4.39	4.12	3.51	4.72	4.88	3.11	2.90	4.46	4.48	3.89	3.04	4.33	5.36	4.88	5.13
12	3.75	4.30	4.01	3.46	4.13	4.53	3.38	3.59	4.92	4.56	3.78	3.77	4.04	5.38	5.65	5.24

SOD

	Passive recovery				Shock wave				Pneumatic compression				PBMT-sMF			
**ID**	Baseline	1h	24h	48h	Baseline	1h	24h	48h	Baseline	1h	24h	48h	Baseline	1h	24h	48h
1	3.13	3.49	2.63	3.16	3.66	3.28	3.65	3.51	3.65	3.09	3.40	3.37	3.33	4.06	4.21	4.12
2	3.60	3.70	2.98	2.98	3.62	3.71	3.27	3.69	3.57	3.54	3.35	3.07	3.13	4.41	4.12	4.04
3	3.97	3.58	3.54	3.12	3.50	3.72	3.16	3.59	3.49	3.46	2.64	2.13	3.97	4.50	4.47	4.55
4	3.62	3.71	4.12	3.98	3.09	3.65	2.78	3.00	3.04	3.24	3.61	3.06	3.92	4.29	4.04	4.48
5	3.01	3.72	2.98	3.02	3.18	3.26	2.61	2.96	3.86	4.36	3.24	2.67	3.53	4.15	4.49	5.28
6	3.48	3.69	3.05	2.79	3.20	3.55	3.56	3.23	3.51	3.53	2.62	2.97	3.02	3.01	4.77	4.40
7	3.16	3.46	3.44	2.91	3.90	4.05	3.03	2.72	3.13	3.79	2.79	3.10	2.95	3.71	3.36	4.76
8	3.29	3.40	2.67	2.86	3.57	3.98	3.73	2.25	3.06	3.30	3.36	2.28	3.62	3.63	4.45	3.95
9	3.73	3.61	3.52	2.80	2.95	3.83	3.45	3.33	4.05	3.64	3.45	3.18	3.12	3.69	3.93	4.34
10	4.06	3.19	3.64	2.52	3.35	3.01	2.92	3.13	3.15	3.06	2.87	3.37	2.95	4.01	4.41	4.09
11	3.81	2.24	3.65	2.57	3.56	3.25	3.96	2.54	3.25	3.03	2.31	2.87	4.19	3.80	4.56	3.96
12	3.53	2.98	2.96	2.71	3.05	3.87	2.75	2.85	3.89	4.14	2.31	2.92	3.29	3.64	4.24	4.77

RPE - R

	PBMT-sMF						Shock Wave						Passive recovery						Pneumatic compression					
**ID**	Baseline	Post *WOD*	Post treatment	1h	24h	48h	Baseline	Post *WOD*	Post treatment	1h	24h	48h	Baseline	Post *WOD*	Post treatment	1h	24h	48h	Baseline	Post *WOD*	Post treatment	1h	24h	48h
1	0	100	5	2	0	0	0	90	5	5	0	0	5	100	0	0	0	0	0	90	10	10	10	3
2	0	100	0	0	0	0	2	90	0	0	0	0	0	90	0	0	0	0	0	90	0	0	0	0
3	0	100	0	0	0	0	1.5	100	0	0	0	0	0	100	20	20	0	0	30	100	25	25	0	0
4	0	80	10	5	0	0	0	85	0	0	0	0	0	95	0	0	0	0	0	100	0	0	0	0
5	20	90	0	0	0	0	0	100	0	0	0	0	0	80	0	0	0	0	0	60	0	0	0	0
6	0	80	10	0	0	0	0	80	0	0	0	0	0	80	0	0	0	0	0	70	0	0	0	0
7	0	95	20	0	0	0	0	60	0	0	0	0	0	95	25	25	10	6	15	90	20	16	2.5	0
8	0	63	0	0	0	0	0	80	10	5	0	0	0	85	10	0	0	0	0	85	0	0	4	0
9	0	60	0	0	0	0	0	70	10	0	0	0	0	70	0	0	0	0	0	80	0	0	0	0
10	15	70	18	15	5	0	0	80	12	10	12	10	1	70	55	55	0	0	12	60	10	5	0	0
11	0	10	0	0	0	0	0	15	0	0	0	0	0	50	5	0	0	0	0	20	0	0	0	0
12	5	95	0	0	0	0	0	90	0	0	0	0	0	98	10	8	0	0	0	96	0	0	0	0

RPE - MI

	PBMT-sMF						Shock Wave						Passive recovery						Pneumatic compression					
**ID**	Baseline	Post *WOD*	Post treatment	1h	24h	48h	Baseline	Post *WOD*	Post treatment	1h	24h	48h	Baseline	Post *WOD*	Post treatment	1h	24h	48h	Baseline	Post *WOD*	Post treatment	1h	24h	48h
1	17	95	35	35	10	4	8	100	30	25	8	6	5	85	15	12	5	3	10	90	40	35	17	5
2	50	90	70	70	50	40	2	90	15	15	15	10	15	90	20	7	15	6	6	90	60	60	50	50
3	20	100	40	40	10	10	0	100	40	30	0	0	10	90	30	30	40	20	30	100	50	50	20	10
4	25	100	40	30	10	8	58	100	75	75	55	30	25	95	40	40	40	40	40	100	50	45	30	20
5	35	75	30	30	35	10	0	75	30	0	0	0	0	45	15	12	0	0	7	70	30	25	20	0
6	15	75	25	25	25	15	0	60	20	15	15	10	0	25	20	10	10	5	0	50	5	5	40	7
7	0	80	30	0	0	0	12	60	15	5	7	15	0	65	60	60	10	6	15	90	25	9	2.5	0
8	0	87	27	15	0	0	0	70	70	80	12	35	0	38	15	15	4	3	3	70	25	23	4	3
9	45	50	30	37	40	30	50	65	50	40	20	20	10	90	20	15	25	20	30	45	40	40	25	23
10	20	90	36	25	23	8	12	100	20	25	30	10	2	87	60	60	30	15	7	100	15	15	6	6
11	5	90	25	20	25	25	12	90	12	12	5	6	1.5	70	70	70	75	60	0	80	15	15	7	1.5
12	12	90	30	25	20	25	20	85	15	9	8	12	12	95	25	25	8	4	10	95	0	8	8	8
