# Supplementary material for: A randomised crossover trial comparing photobiomodulation therapy with other recovery strategies in CrossFit athletes
Source: PLoS One. 2026 May 22;21(5):e0349880. doi: 10.1371/journal.pone.0349880 (PMC13196929; doi:10.1371/journal.pone.0349880)

**Host Institution:** Universidade Nove de Julho – UNINOVE

**Project Title:** Isolated and Combined Effects of Different Post-Exercise Recovery Strategies in

Cross Training Athletes: A Randomized, Controlled, and Blinded Clinical Trial

**Student:** Paulo Henrique Gusmão Nogueira Martins

**Supervisor:** Dr. Ernesto Cesar Pinto Leal Junior

**ABSTRACT**

Cross Training is a high-intensity exercise modality, in which the most demanding part of training and competition is called the WOD (workout of the day). Competitions consist of multiple WODs performed in sequence and across consecutive days, which may lead to muscle fatigue and increase athletes’ susceptibility to injuries. In some sports, therapeutic devices have been used to accelerate muscle recovery or modulate the damage caused by strenuous exercise, such as photobiomodulation therapy combined with a static magnetic field (PBMT-sMF), extracorporeal shock wave therapy (ESWT), and intermittent pneumatic compression (IPC). However, it is still unclear which modality is most effective in promoting faster and more efficient muscle recovery in Cross Training athletes. Therefore, the aim of this study is to compare the isolated and combined effects of three different therapeutic modalities—PBMT-sMF, ESWT, and IPC—on muscle recovery in Cross Training athletes, as well as to investigate whether there will be adverse effects related to physiological recovery. To this end, a randomized, controlled, crossover, blinded clinical trial will be conducted. Male Cross Training athletes aged 18 to 36 years will voluntarily participate and will be randomly allocated to receive, in crossover order, the four interventions (control/passive recovery, PBMT-sMF, ESWT, and IPC) across four weeks of procedures. Muscle recovery will be assessed at baseline (prior to any intervention) and at 1 h, 24 h, and 48 h following a WOD designed to induce fatigue. Assessments will include the primary outcome: performance in a functional test; and secondary outcomes: subjective perception of effort/fatigue measured by the CR-100 scale, physiological variables through analysis of muscle damage via lactate dehydrogenase (LDH) levels, as well as thiobarbituric acid reactive substances (TBARS), carbonylated proteins, catalase (CAT), and superoxide dismutase (SOD) activity to assess oxidative stress. Data will be analyzed both in absolute values and as percentage changes from baseline (pre-exercise) values. The Kolmogorov-Smirnov test will be used to verify data normality. Statistical significance will be set at p < 0.05.

**Keywords:** Photobiomodulation Therapy, Shock Wave Therapy, Intermittent Pneumatic Compression, Fatigue, Performance, Recovery.

**BACKGROUND**

Cross Training emerged in the early 2000s as a method of strength and conditioning training (Meyer et al., 2017). Training is based on the combination of functional movements, constantly varied and performed at high intensity, called WOD (Workout of the Day) (Butcher et al., 2015). A WOD consists of a wide range of movements, such as weightlifting exercises (deadlift, bench press, squats), Olympic lifts (snatch and clean and jerk), gymnastics and calisthenics exercises, such as pull-ups, push-ups, sit-ups, and burpees (Butcher et al., 2015; Drum et al., 2017). In addition, Cross Training includes cardiorespiratory stimuli such as running, cycling, rowing, jumping rope, and other plyometric activities (Drum et al., 2017). These stimuli are often combined and performed at high intensity, with fast, successive repetitions and with little or no recovery time (Tibana et al., 2016).

WODs are scored and often involve competitive elements among participants, a feature that has led this method of functional exercise to be recognized as the “Sport of Fitness” (Hak et al., 2013). The official Cross Training competition, known as the CrossFit® Games, was first held in 2007 with 70 athletes, and in recent years participation has expanded to more than 324,000 athletes worldwide (https://games.crossfit.com/history-of-the-games). In this competition, winners are recognized as the best-conditioned athletes in the world, “The Fittest on Earth” (Dawson, 2015).

According to Claudino et al. (2018), Cross Training is considered an excellent option of HIIT (High-Intensity Interval Training). HIIT is a type of interval training at high intensity that improves both anaerobic and aerobic energy supply systems (Tabata et al., 1996; Zhang et al., 2017). When applied to different sports and in Cross Training competitions, HIIT effectively overloads both anaerobic and aerobic energy systems, resulting in improved athletic performance (Tabata et al., 1997; Jagim et al., 2015). However, Cross Training as a sport differs from HIIT by including gymnastic and weightlifting elements, and by reducing or eliminating rest periods (Meyer et al., 2017; Kliszczewicz et al., 2015). As observed in traditional competitions, the main goal of WODs is to complete a specific number of repetitions and exercises in the shortest possible time (Kliszczewicz et al., 2015). Thus, since athletes are often subjected to several WODs on the same day and on consecutive days of competition, they must cope with fatigue and the resulting muscle recovery demands (Hak et al., 2013).

This high physical demand required in intense exercise has raised concerns in the scientific community, as confirmed by several publications investigating the relationship between Cross Training practice and the risk of injuries (Hak et al., 2013; Weisenthal et al., 2014; Sprey et al., 2016; Summitt et al., 2016; Maté-Muñoz et al., 2017). Weisenthal et al. (2014) concluded that fatigue causes alterations in movement biomechanics, increasing injury risk. According to Hak et al. (2013), in their analysis of the nature and prevalence of injuries in Cross Training practitioners, the loss of technique during extreme fatigue is associated with lumbar spine injuries. Maté-Muñoz et al. (2017) added that many exercises used in Cross Training require advanced technique as well as high sustained power output. Therefore, they can induce considerable fatigue and lead to injuries in subsequent training sessions. The fatigue process leads to a decline in physical performance and, in some cases, to injuries (Andersson et al., 2008; Ispirlidis et al., 2008; Nédélec et al., 2012). Thus, it can be inferred that the risk of injury is closely related to fatigue generated by the high intensity required during WOD performance (Hak et al., 2013; Weisenthal et al., 2014; Maté-Muñoz et al., 2017).

The development of muscle fatigue is a complex and multifactorial process that may be associated with muscle oxidative stress caused by increased production of reactive oxygen species (ROS) after strenuous exercise (Reid et al., 1992; Vollaard et al., 2005; Reid et al., 2016). To investigate fatigue during two consecutive Cross Training sessions, Tibana et al. (2016) analyzed physiological responses of blood markers such as creatine kinase (CK), interleukins 6 and 10 (IL-6, IL-10), osteoprotegerin, lactate, and glucose. Based on their results, the authors highlighted the importance of lower-intensity sessions and/or rest days to help minimize immune disturbances and possible causes of fatigue (Tibana et al., 2016). Similarly, Heavens et al. (2014) analyzed the effects of high-intensity training on CK, IL-6, myoglobin, and testosterone markers and concluded that training intensity should be individually scaled to minimize injury risk. They also pointed out the importance of recovery periods and rest days between high-intensity training sessions (Heavens et al., 2014). Some studies assessing blood markers after strenuous exercise suggest that the increase of these cytokines is associated with muscle damage and elevated neutrophil count during muscle recovery (Yamada et al., 1985; Bruunsgaard et al., 1997).

Muscle recovery strategies involve the implementation of a single or combined technique to enhance and accelerate recovery after exercise, better preparing the athlete for the next activity and reducing the risk of injury (Dupont et al., 2010; Pinto et al., 2016). Sports-related studies have sought tools to minimize exercise-induced damage, optimize training intervals, increase performance, and prevent injuries (Kellmann et al., 2010). According to Kellmann et al. (2010), the difference between success and failure in modern sports is directly related to recovery effectiveness. Supporting this claim, Feito et al. (2018) indicated that rapid and efficient muscle recovery capacity is an important factor for better performance in Cross Training. Therefore, there is evidence highlighting the relevance of investigating resources that promote muscle recovery according to the physical demands of the sport. Historically and clinically, several therapeutic resources have been employed to recover from muscle fatigue and, consequently, enhance performance in athletes, regardless of the strength of scientific evidence, and they are widely applied in clinical practice (Altarriba-Bartes et al., 2020).

Some therapeutic modalities are commonly used for muscle recovery in sports practice, such as massage (Hoffman et al., 2016), cryotherapy (Micheletti et al., 2019), intermittent pneumatic compression – IPC (Hoffman et al., 2016), extracorporeal shock wave therapy – ESWT (Fleckenstein et al., 2017), and photobiomodulation therapy – PBMT (Tomazoni et al., 2019; De Marchi et al., 2019). Recent studies have evaluated IPC, ESWT, and PBMT, and have suggested positive outcomes (Hoffman et al., 2016; Tomazoni et al., 2019; De Marchi et al., 2019).

IPC, used both by athletes and in rehabilitation clinics, consists of inflatable chambers/boots that surround the limb and sequentially inflate with air to apply pressure (Hoffman et al., 2016; Martin et al., 2015; Haun et al., 2017; Overmayer et al., 2018). Studies suggest that negative pressure can improve recovery and reduce muscle soreness after strenuous exercise (Hoffman et al., 2016; Martin et al., 2015). IPC works by improving venous return at a circulatory level, theoretically enhancing muscle blood flow, increasing tissue oxygenation, and positively influencing muscle recovery (Zuj et al., 2019). Furthermore, IPC has shown satisfactory results in various vascular dysfunctions such as lymphedema, severe chronic venous insufficiency, peripheral arterial occlusive disease, and thrombosis prophylaxis (Schwahn et al., 2018). However, some studies report inconclusive results regarding IPC for reducing fatigue and improving muscle performance, which may be related to the lack of standardized parameters for this purpose, since parameters used vary widely (Hoffman et al., 2016; Cochrane et al., 2013; Martin et al., 2015; Overmayer & Driller, 2017).

With effective results in systematic reviews for a variety of musculoskeletal clinical conditions, ESWT has been emerging as a potential new treatment option (Schmitz et al., 2015; Alabbad et al., 2020). ESWT delivers three-dimensional pressure pulses concentrated in microseconds with peak pressures of 35–120 MPa, applied to small focal areas of 2–8 mm in diameter, perpendicularly to the tissue (Alabbad et al., 2020). Its mechanisms of action include effects on tissue calcifications, alterations in cellular activity through cavitation, acoustic microstreaming, changes in cell membrane permeability, and effects on nociceptors through hyperstimulation, blocking the pain gate-control mechanism (Speed et al., 2014). Satisfactory effects have been found in stimulating skeletal muscle tissue regeneration and accelerating repair processes in acute muscle injury, indicating increased proliferation and differentiation rates of satellite cells (Zissler et al., 2017). Moreover, ESWT has demonstrated positive results in clinical studies for injury rehabilitation, muscle recovery in athletes (Speed et al., 2014; Fleckenstein et al., 2017), and reduction of muscle soreness (Fleckenstein et al., 2017; Notarnicola et al., 2018; Taheri et al., 2016; Jeon et al., 2012). Fleckenstein et al. (2017) examined the effects of a single ESWT application on delayed onset muscle soreness induced by eccentric exercise and observed clinically relevant effects in pain relief and increased muscle strength. Similarly, Notarnicola et al. (2018) demonstrated significant increases in muscle elasticity, tone, and fiber recruitment with ESWT applied to the quadriceps. ESWT has also shown positive outcomes in musculoskeletal dysfunctions such as epicondylitis, subacromial pain syndrome, plantar fasciitis, and tendinopathies (Schmitz et al., 2015). These results point to ESWT as a possible tool to help delay fatigue and improve muscle performance, although few studies have demonstrated its effects on muscle recovery after strenuous exercise in athletes.

Recent studies on PBMT have shown satisfactory results for both reducing fatigue markers and enhancing performance and muscle strength (Tomazoni et al., 2019; De Marchi et al., 2019; Pinto et al., 2016; Vanin et al., 2018; Leal-Junior et al., 2019; Baroni et al., 2010; Antonialli et al., 2014; De Paiva et al., 2016; Vanin et al., 2016). Results suggest attenuation of delayed-onset muscle soreness and smaller increases in blood markers of muscle damage when PBMT is used compared to control groups (Baroni et al., 2010; De Paiva et al., 2016). Its mechanism of action is related to enhanced electron transfer, consequently activating mitochondrial respiratory chains and increasing mitochondrial adenosine triphosphate (ATP) production (Miranda et al., 2018; Wang et al., 2018). Tomazoni et al. (2019) showed that pre-exercise PBMT, as a standalone therapy, was able to improve different functional aspects related to athletic performance and biochemical markers of muscle damage and inflammation in elite athletes. Additionally, PBMT demonstrated antioxidant effects, reducing exercise-induced oxidative stress and promoting ergogenic and protective effects on skeletal muscles (Tomazoni et al., 2019).

Furthermore, studies have reported positive outcomes using PBMT combined with a static magnetic field – PBMT-sMF (Antonialli et al., 2014; De Paiva et al., 2016). The magnetic field enhances ATP production and helps reduce oxidative stress, thereby potentiating the effects of PBMT (Friedmann et al., 2019; Wang et al., 2018; Coballase-Urrutia et al., 2018). According to Pinto et al. (2016), in a field test with rugby athletes, PBMT-sMF improved performance and promoted faster muscle recovery.

However, evidence on the effectiveness of some of these recovery strategies remains conflicting (Brown et al., 2017; Poppendieck et al., 2016). Considering the high-intensity effort required in Cross Training, the use of resources that support the muscle recovery process is of great value, as it can ensure athletes are fit to perform subsequent WODs with reduced injury risk. Thus, advancing the development and understanding of therapies or resources tailored to the recovery needs of Cross Training athletes is highly important for optimal performance in this sport. The present study seeks to compare therapeutic resources frequently employed in clinical practice in order to determine the most suitable for muscle recovery associated with Cross Training practice.

**Hypothesis**

Based on previous studies conducted by different research groups, in laboratory and field settings, we hypothesize that the use of therapeutic resources will favor muscle recovery optimization in Cross Training athletes after the completion of a WOD, which is considered a strenuous exercise.

**Objectives**

**General Objective:**

To compare the effects of three different therapeutic resources—PBMT-sMF, IPC, and ESWT—on muscle recovery in Cross Training participants.

**Specific Objectives:**

- To analyze the effects of therapeutic resources on performance-related variables by assessing muscle recovery capacity through functional evaluation.
- To evaluate the effects of therapeutic resources on biochemical markers related to muscle recovery and oxidative stress.

**METHODS**

**Study Design and Ethics**

This randomized, controlled, crossover clinical trial with blinded outcome assessors will be prospectively registered at ClinicalTrials.gov. The study will be submitted to the Ethics Committee of Universidade Nove de Julho and conducted in accordance with the Declaration of Helsinki. Written informed consent will be obtained.

**Participants**

The trial will be conducted in São José dos Campos, Brazil. Healthy male amateur Cross Training athletes aged between 18 and 35 years, who have been training at least four times per week for a minimum of one year, will be included. Exclusion criteria will include pharmacological use or recent musculoskeletal injuries. In addition, individuals who sustain musculoskeletal or joint injuries during the study will also be excluded.

**Interventions**

All interventions will be applied five minutes after the WOD and will last 30 minutes, except for PBMT-sMF, which will be applied for 32 minutes.

- **Passive Recovery (PR):** Participants will remain at rest in the supine position throughout the intervention period, serving as the control condition (Gill et al., 2006).
- **Intermittent Pneumatic Compression (IPC):** Sequential compression will be performed using pneumatic boots (model 701 RA, Recovery Pump LLC, Concordville, PA, USA), positioned on both lower limbs. The device will apply a pressure of 80–90 mmHg in four inflatable chambers arranged linearly from the feet to the hips. Each chamber will be sequentially inflated for approximately 8 to 12 seconds, followed by a partial deflation period of 15 seconds, with the complete inflation cycle lasting 30 to 40 seconds (Martins et al., 2015).
- **Extracorporeal Shock Wave Therapy (ESWT):** The intervention will be administered using the Shock Wave Therapy BTL-6000 device (BTL Industries Ltd., United Kingdom), according to the manufacturer’s instructions. Each session will involve 2,000 pulses per region (6,000 pulses per lower limb), with an energy flux density of 0.03 mJ/mm², a frequency of 10 Hz, and a pressure of 1.5 bar (Fleckenstein et al., 2017). A 15 mm diameter acoustic wave transmitter will be applied directly to the skin with conductive gel to optimize coupling and energy distribution (Fleckenstein et al., 2017). ESWT will be targeted at the knee extensor/hip flexor muscles (rectus femoris, vastus lateralis, vastus medialis), knee flexor/hip extensor muscles (hamstrings), and plantar flexor muscles (gastrocnemius) in both lower limbs. Application sites and protocol will follow a previous study and manufacturer guidelines to ensure optimal effectiveness (Fleckenstein et al., 2017).
- **Photobiomodulation Therapy Combined with Static Magnetic Field (PBMT-sMF):** This intervention will be performed using a Multi Radiance Medical device (Solon, OH, USA) equipped with a cluster probe that emits a negligible amount of heat (Grandinétti et al., 2015). The device will contain 20 diodes, distributed as follows: four diodes emitting at 905 nm (1.25 mW average power, 50 W peak power per diode), eight diodes emitting at 850 nm (40 mW average power per diode), and eight diodes emitting at 633 nm (25 mW average power per diode). PBMT-sMF will be applied to eight specific sites in both lower limbs, targeting: four sites in the knee extensor/hip flexor muscles (rectus femoris, vastus lateralis, and vastus medialis); three sites in the knee flexor/hip extensor muscles (hamstrings); and one site in the plantar flexor muscles (gastrocnemius). The parameters for PBMT-sMF will be selected based on previous research to ensure consistency and therapeutic effectiveness (Vanin et al., 2018; Pinto et al., 2022; Antonialli et al., 2014). A full list of PBMT-sMF parameters will be provided in Table 1.

Table 1. PBMT-sMF parameters.

|  | Lasers | Red LEDs | Infrared LEDs |
| --- | --- | --- | --- |
| Number of diodes | 4 | 8 | 8 |
| Wavelength (nm) | 905 | 633 | 850 |
| Frequency (Hz) | 250 | 2 | 250 |
| Peak power (W) | 50 | - | - |
| Average optical output (mW)- each | 1.25 | 25 | 40 |
| Power density (mW/cm^2^) - each | 3.91 | 29.41 | 71.23 |
| Energy density (J/cm^2^) - each | 0.50*; 0.44**; 0.44*** | 3.79*; 3.39**; 3.39*** | 9.21*; 8.21**; 8.21*** |
| Dose (J) - each | 0.16*; 0.14**; 0.14*** | 3.22*; 2.88**; 2.88*** | 5.16*; 4.60**; 4.60*** |
| Spot size of diode (cm^2^) - each | 0.32 | 0.85 | 0.56 |
| Magnetic field (mT) | 110 | | |
| Irradiation time per site (sec) | 129*; 115**; 115*** | | |
| Total dose per site (J) | 67.68*; 60.76**; 60.76*** | | |
| Total dose applied per lower limb (J) | 270.72*; 182.28**; 60.76*** | | |
| Aperture of device | 33 | | |
| Application mode | Direct skin contacts and slight pressure | | |

* Knee extensors, ** knee flexors, *** plantar flexors.

**Blinding and Randomization**

Outcomes will be assessed by an independent evaluator who will be blinded to group allocation. Intervention sequences will be randomized (1:1:1:1) via www.randomization.org by a researcher not involved in other phases of the study. Each participant will complete all four interventions across weekly sessions with seven-day washout periods. Allocation will be concealed using sealed, numbered, opaque envelopes.

The intervention orders will be:

- Sequence 1: PR → PBMT-sMF → ESWT → IPC
- Sequence 2: PBMT-sMF → ESWT → IPC → PR
- Sequence 3: ESWT → IPC → PR → PBMT-sMF
- Sequence 4: IPC → PR → PBMT-sMF → ESWT

**Study Sessions and Exercise Protocol**

The trial will comprise four sessions with seven-day washouts. Procedures will remain identical except for the randomized intervention applied. Assessments will occur before and after each WOD.

- Exercise Protocol – WOD: To minimize circadian and external influences, participants will perform the WOD at the same time in all sessions and will avoid physical activity from 24 h pre-WOD to 48 h post-WOD. The WOD will follow the Cross Training 21-15-9 scheme (three rounds, no rest), aiming for the fastest possible completion. Repetitions will be monitored by an independent assessor.

The exercises will include:

1. Assault AirBike Calories: Participants will use a standardized AirBike model, pedaling until they reach the target calorie count required for each round.
2. Hang Squat Clean: The exercise will start with the participant holding the barbell in the hang position (above the knees) with arms fully extended. From this position, the barbell will be cleaned to the shoulders while the hips descend below the knees. A repetition will be validated when full hip and knee extension is achieved with the bar resting on the shoulders.
3. Box Jump Over: Participants will perform a two-foot jump onto a 60.69-cm (24-inch) box, ensuring both feet contact the top surface before jumping down to the opposite side.

**Outcomes**

The primary outcome will be the change in performance on the functional test, assessed 1-hour post-WOD. Secondary outcomes will include: change in functional test performance at 24 and 48 hours post-WOD; biochemical markers of muscle damage and oxidative stress; antioxidant activity; subjective perception of exertion (assessed at 1, 24, and 48 hours post-WOD); and satisfaction with the intervention (assessed at 48 hours post-WOD).

- Vertical Jump Performance: Vertical jump performance will be measured using the countermovement jump (CMJ) height test (Balsalobre-Fernández et al., 2015). During the test, participants will stand with feet shoulder-width apart and hands on the hips. They will perform a controlled squat to ~90° of knee flexion followed by an explosive vertical jump. Upon landing, they will flex the hips, knees, and ankles to absorb impact. A smartphone camera, positioned 1.5 m away, will record three maximal-effort CMJ attempts (Balsalobre-Fernández et al., 2015). Jump height will be analyzed using the JumPo 2 app (iOS), the only validated tool available at the time. The highest recorded jump will be used for analysis.
- Muscle Damage and Oxidative Stress Markers: Muscle damage will be assessed via lactate dehydrogenase (LDH) levels (De Oliveira et al., 2017). Oxidative stress and antioxidant activity will be assessed using thiobarbituric acid reactive substances (TBARS), carbonylated proteins, catalase (CAT), and superoxide dismutase (SOD) (Pinto et al., 2022). Blood samples (5 mL) will be drawn from the anterior cubital vein and stored at −80 °C for later analysis. LDH activity will be quantified by spectrophotometry using specific reagent kits (Labtest®, Minas Gerais, Brazil) as an indirect marker of muscle damage. TBARS, carbonylated proteins, CAT, and SOD will be analyzed as biomarkers of oxidative stress and antioxidant defense following previously established spectrophotometric protocols (Pinto et al., 2022). Each biochemical analysis will be conducted in triplicate, and the mean value will be used for statistical interpretation.
- Subjective Perception of Exertion: Perceived exertion will be assessed using the Rate of Perceived Exertion (RPE) scale (Fanchini et al., Weston et al., 2015). Participants will rate effort in two domains:
- RPE-MI (Muscular Intensity): perceived lower-limb fatigue.
- RPE-R (Respiratory Effort): perceived cardiorespiratory fatigue.
  Volunteers will complete two identical RPE scales ranging from 0 to 100, corresponding to muscular and cardiorespiratory effort.
- Satisfaction with the Intervention: Satisfaction will be assessed 48 hours post-WOD using a 5-point Likert scale: (1) Very unsatisfied, (2) Unsatisfied, (3) Neutral, (4) Satisfied, (5) Very satisfied. For analysis, responses will be dichotomized as satisfied (“satisfied” and “very satisfied”) versus not satisfied (“neutral,” “unsatisfied,” and “very unsatisfied”).

**Statistical Analysis**

To the best of our knowledge, no prior study will have compared PBMT-sMF, IPC, ESWT, and passive recovery in Cross Training athletes. Therefore, sample size will be calculated based on CMJ performance data from the first session, following a previously described method (Pinto et al., 2022). This calculation will be conducted by a blinded researcher and will use percentage changes in CMJ at 1-hour post-WOD (primary outcome). An 80% statistical power (α = 0.05; ANOVA) will be considered, and the WebPower (https://webpower.psychstat.org/models/means03/effectsize.php) and Statistics Kingdom (https://www.statskingdom.com/sample_size_regression.html) tools will be used for the computation.

Statistical analyses will follow the intention-to-treat principle (Elkins, 2015). The statistician will remain blinded to allocation to minimize bias. Data normality will be assessed using the Shapiro–Wilk test. Differences between interventions will be evaluated using repeated-measures ANOVA with Bonferroni post hoc tests. Both absolute values and percentage changes from baseline will be analyzed. Results will be presented as mean ± SD (tables) and mean ± SEM (figures). Fisher’s exact test will be used to compare satisfaction proportions across interventions. Statistical significance will be set at p < 0.05.

**REFERENCES**

Andersson, H.; Raastad, T.; Nilsson, J.; Paulsen, G.; Garthe, I. and Kadi, F. “Neuromuscular fatigue and recovery in elite female soccer: effects of active recovery,” Medicine and Science in Sports and Exercise, vol. 40, no. 2, pp. 372–380, 2008.

Aksenov MY, Markesberya WR. Changes in thiol content and expression of glutathione redox system genes in the hippocampus and cerebellum in Alzheimer’s disease. Neurosci Lett 2001;302:141-5. 21.

Al-Abbad, H., Allen, S., Morris, S. et al. The Effects of shockwave therapy on musculoskeletal conditions based on changes in imaging: a systematic review and meta-analysis with meta regression. BMC Musculoskelet Disord 21, 275 (2020).

Altarriba-Bartes A, Peña J, Vicens-Bordas J, Milà-Villaroel R, Calleja-González J (2020) Estratégias de recuperação pós-competição em jogadores de futebol de elite. Efeitos no desempenho: uma revisão sistemática e meta-análise. PLoS ONE 15 (10): e0240135. https://doi.org/10.1371/journal.pone.0240135

Antonialli FC, De Marchi T, Tomazoni SS, et al. Phototherapy in skeletal muscle performance and recovery after exercise: effect of combination of super-pulsed laser and light-emitting diodes. Lasers Med Sci. 2014; 29: 1967-1976.

Balsalobre-Fernández C, Glaister M, Lockey RA. The validity and reliability of an iPhone app for measuring vertical jump performance. J Sports Sci. 2015;33: 1574-1579.

Bannister JV, Calabrese L. Assay for SOD. Meth Biochem 1987;32:279-312.

Baroni BM, Leal Junior EC, De Marchi T, Lopes AL, Salvador M, Vaz MA. (2010) Low level laser therapy before eccentric exercise reduces muscle damage markers in humans. Eur J Appl Physiol. 110: 789-96.

Borg E, Borg G. (2002) A comparison of AME and CR100 for scaling perceived exertion. Acta Psychol (Amst). Feb;109(2):157-75. PubMed PMID: 11820425.

Brown, F.; Gissane, C.; Howatson, G.; vanSomeren, K.; Pedlar, C. and Hill, J. “Compression garments and recovery from exercise: a meta-analysis,” Sports Medicine, vol. 47, no. 11, pp. 2245– 2267, 2017.

Bruunsgaard H, Galbo H, Halkjaer-Kristensen J, Johansen TL, MacLean DA, and Pedersen BK. Exercise-induced increase in serum interleukin-6 in humans is related to muscle damage. J Physiol 499: 833–841, 1997.

Butcher SJ, Neyedly TJ, Horvey KJ, Benko CR. Do physiological measures predict selected CrossFit^®^ benchmark performance? Open Access J Sports Med. 2015 Jul 31;6:241-7.

Cayton T, Harwood AE, Smith GE, Totty JP, Carradice D, Chetter IC. (2017) Extracorporeal shockwave therapy for the treatment of lower limb intermittent claudication: study protocol for a randomised controlled trial (the SHOCKWAVE 1 trial). Trials. 6;18(1):104.

Claudino JG, Gabbett TJ, Bourgeois F, Souza HS, Miranda RC, Mezêncio B, Soncin R, Cardoso Filho CA, Bottaro M, Hernandez AJ, Amadio AC, Serrão JC. (2018) Crossfit Overview: Systematic Review and Meta-analysis. Sports Med Open. Feb 26;4(1):11.

Cochrane DJ, Booker HR, Mundel T, Barnes MJ. (2013) Does intermittent pneumatic leg compression enhance muscle recovery after strenuous eccentric exercise? Int J Sports Med. Nov;34(11):969-74.

Coballase-Urrutia E, Navarro L, Ortiz JL, Verdugo-Díaz L, Gallardo JM, Hernández ME, Estrada-Rojo F. (2018) Static magnetic fields modulate the response of different oxidative stress markers in a restraint stress model animal. Biomed Res Int 3960408.

Dawson, M. C. (2015). Crossfit: Fitness cult or reinventive institution? International Review for the Sociology of Sport, 52(3), 361–379.

De Marchi T, Leal-Junior ECP, Lando KC, Cimadon F, Vanin AA, da Rosa DP, Salvador M. (2019) Photobiomodulation therapy before futsal matches improves the staying time of athletes in the court and accelerates post-exercise recovery. Lasers Med Sci. Feb;34(1):139-148.

De Oliveira AR, Vanin AA, Tomazoni SS, et al. Pre-exercise infrared photobiomodulation therapy (810 nm) in skeletal muscle performance and postexercise recovery in humans: what is the optimal power output? Photomed Laser Surg. 2017;35: 595-603.

De Paiva PV, Tomazoni SS, Johnson DS, Vanin AA, Albuquerque-Pontes GM, Machado CSM, Casalechi HL, De Carvalho PTC, Leal-Junior ECP . (2016) Photobiomodulation therapy (TFBM) and/or cryotherapy in skeletal muscle restitution, what is better? A randomized, double-blinded, placebo-controlled clinical trial. Lasers in Medical Science. 31,1925-1933.

Draper HH, Hadley M. Malondialdehyde determination as index of lipid peroxidation. Meth Enzymol 1990;186:421-31. 19.

Dornelles MP, Fritsch CG, Sonda FC, Johnson DS, Leal-Junior ECP, Vaz MA, Baroni BM. Photobiomodulation therapy as a tool to prevent hamstring strain injuries by reducing soccer-induced fatigue on hamstring muscles. Lasers Med Sci. 2019 Aug;34(6):1177-1184. doi: 10.1007/s10103-018-02709-w. Epub 2019 Jan 3. PMID: 30607719.

Drum SN, Bellovary BN, Jensen RL, Moore MT, Donath L. Perceived demands and postexercise physical dysfunction in CrossFit® compared to an ACSM based training session. J Sports Med Phys Fitness. 2017 May;57(5):604-609.

Dupont, G.; Nedelec, M.; McCall, A.; McCormack, D.; Berthoin, S. and Wisløff, U. “Effect of 2 soccer matches in a week on physical performance and injury rate,” The American Journal of Sports Medicine, vol. 38, no. 9, pp. 1752–1758, 2010.

Elkins MR, Moseley AM. Intention-to-treat analysis. J Physiother. 2015;61: 165-167.

Feito Y, Heinrich KM, Butcher SJ, Poston WSC. High-Intensity Functional Training (HIFT): Definition and Research Implications for Improved Fitness. Sports (Basel). 2018 Aug 7;6(3):76.

Figueiredo, M.; Simao, P P; Pereira, Beethoven, M. A.  and  Penha-Silva, N.(2008) Eficácia da compressão pneumática intermitente (CPI) nos membros inferiores sobre o fluxo sanguíneo das veias femorais comuns. J. vasc. bras, vol.7, n.4, pp.321-324.

Fleckenstein J, Friton M, Himmelreich H, et al. Effect of a single administration of focused extracorporeal shock wave in the relief of delayed-onset muscle soreness: results of a partially blinded randomized controlled trial. Arch Phys Med Rehabil. 2017;98: 923-930.

Fanchini M, Ferraresi I, Modena R et al. Use of CR100 Scale for session rating of perceived exertion in soccer and its interchangeability with the CR10. Int J Sports Physiol Perform. 2016;11: 388-392.

Friedmann H, Lipovsky A, Nitzan Y, Lubart R. (2019) Combined magnetic and pulsed laser fields produce synergistic acceleration of cellular electron transfer. Laser Ther. 18(3): 137-4.

Gill ND, Beaven CM, Cook C. Effectiveness of post-match recovery strategies in rugby players. Br J Sports Med. 2006;40: 260-263.

Grandinétti Vdos S, Miranda EF, Johnson DS, et al. The thermal impact of phototherapy with concurrent super-pulsed lasers and red and infrared LEDs on human skin. Lasers Med Sci. 2015;30: 1575-1581.

Haifeng Zhang, Tom K. Tong, Weifeng Qiu, Xu Zhang, Shi Zhou, Yang Liu, Yuxiu He J. Comparable Effects of High-Intensity Interval Training and Prolonged Continuous Exercise Training on Abdominal Visceral Fat Reduction in Obese Young Women Diabetes Res. 2017; 2017: 5071740

Hak PT, Hodzovic E, Hickey B. The nature and prevalence of injury during Crossfit training. J Strength Cond Res. 2013 Nov 22.

Harwood AE, Green J, Cayton T, Raza A, Wallace T, Carradice D, Chetter IC, Smith GE. (2018) A feasibility double-blind randomized placebo-controlled trial of extracorporeal shockwave therapy as a novel treatment for intermittent claudication. J Vasc Surg. 67(2):514-521.e2.

Haun CT, Roberts MD, Romero MA, Osburn SC, Mobley CB, Anderson RG, Goodlett MD, Pascoe DD, Martin JS. (2017) Does external pneumatic compression treatment between bouts of overreaching resistance training sessions exert differential effects on molecular signaling and performance-related variables compared to passive recovery? An exploratory study. PLoS One. Jun 29;12(6):e0180429

Heavens, K. R., Szivak, T. K., Hooper, D. R., Dunn-Lewis, C., Comstock, B. A., Flanagan, S. D., et al. (2014). The effects of high intensity short rest resistance exercise on muscle damage markers in men and women. J. Strength Cond. Res.28, 1041–1049.

Hoffman MD, Badowski N, Chin J, Stuempfle KJ. A Randomized Controlled Trial of Massage and Pneumatic Compression for Ultramarathon Recovery. J Orthop Sports Phys Ther. 2016 May;46(5):320-6. doi: 10.2519/jospt.2016.6455. Epub 2016 Mar 23. PubMed PMID: 27011305.

Ispirlidis, I.; Fatouros, I. G.; Jamurtas, A. Z. et al., “Time-course of changes in inflammatory and performance responses fol- lowing a soccer game,” Clinical Journal of Sport Medicine, vol. 18, no. 5, pp. 423–431, 2008.

Jagim AR, Rader O, Jones MT, Oliver JM. The physical demands of multi-modal training competitions and their relationship to measures of performance. J Strength Cond Res. 2015.

Jeon JH, Jung YJ, Lee JY, Choi JS, Mun JH, Park WY, Seo CH, Jang KU. (2012) The effect of extracorporeal shock wave therapy on myofascial pain syndrome. Ann Rehabil Med. Oct;36(5):665-74.

Joondeph SA, Joondeph BC. (2013) Retinal detachment due to Crossfit training injury. Case Rep Ophthalmol Med. 2013: 189837

Karaoglan I, Pehlivan S, Namiduru M, Pehlivan M, Kilinçarslan C, Balkan Y, Baydar I. (2009) TNF-alpha, TGF-beta, IL-10, IL-6 and IFN-gamma gene polymorphisms as risk factors for brucellosis. New Microbiol. Apr;32(2):173-8.

Kellmann, M. Prevenindo overtraining em atletas em esportes de alta intensidade e monitoramento de estresse / recuperação. Scand J Med Sci Sports. 2010; 20: 95–102.

Kliszczewicz, B., Snarr, RL., and Esco, M.. Metabolic and cardiovascular response to the CrossFit workout ‘Cindy’: A pilot study. J Sport Human Perf 2014;2(2):1-9.

Kliszczewicz B., John QC, Daniel BL, Gretchen OD, Michael ER, Kyle TJ Exercício agudo e estresse oxidativo: CrossFit ^TM^ vs. luta em esteira. *J. Hum. Kinet.*2015; 47 : 81–90. doi: 10.1515 / hukin-2015-0064.

Kraemer WJ, Bush JA, Wickham RB, Denegar CR, Gómez AL, Gotshalk LA, Duncan ND, Volek JS, Putukian M, Sebastianelli WJ. Influence of compression therapy on symptoms following soft tissue injury from maximal eccentric exercise. J Orthop Sports Phys Ther. 2001 Jun;31(6):282-90. PubMed PMID: 11411623.

Leal-Junior ECP, Lopes-Martins RÁB, Bjordal JM. (2019) Clinical and scientific recommendations for the use of photobiomodulation therapy in exercise performance enhancement and post-exercise recovery: current evidence and future directions. Braz J Phys Ther. pii: S1413-3555(18) 31021-9.

Levine RL, Garland D, Oliver CN, Amici A, Climent I, Lenz AG, et al. Determination of carbonyl content in oxidatively modified proteins. Meth Enzymol 1990;186:464-78. 20.

Maté-Muñoz JL, Lougedo JH, Barba M, Garcı ´a-Ferna´ndez P, Garnacho-Castaño MV, Domınguez R (2017) Muscular fatigue in response to different modalities of Crossfit sessions. PLoS ONE 12(7): e0181855.

Martin JS, Friedenreich ZD, Borges AR, Roberts MD. (2015) Acute Effects of Peristaltic Pneumatic Compression on Repeated Anaerobic Exercise Performance and Blood Lactate Clearance. J Strength Cond Res. Oct; 29(10) : 2900-6.

Martin JS, Friedenreich, ZD, Borges AR, et al. Preconditioning with peristaltic external pneumatic compression does not acutely improve repeated Wingate performance nor does it alter blood lactate concentrations during passive recovery compared with sham. Appl Physiol Nutr Metab. 2015;40: 1214-7.

Meyer J, Morrison J, Zuniga J. The Benefits and Risks of CrossFit: A Systematic Review. Workplace Health Saf. 2017 Dec;65(12):612-618.

Micheletti JK, Vanderlei FM, Machado AF, de Almeida AC, Nakamura FY, Netto Junior J, Pastre CM. (2019) A New Mathematical Approach to Explore the Post-exercise Recovery Process and Its Applicability in a Cold Water Immersion Protocol. J Strength Cond Res. May;33(5):1266-1275.

Miranda EF, Tomazoni SS, de Paiva PRV, Pinto HD, Smith D, Santos LA, de Tarso Camillo de Carvalho P, Leal-Junior ECP.(2018) When is the best moment to apply photobiomodulation therapy (PBMT) when associated to a treadmill endurance-training program? A randomized, triple-blinded, placebo-controlled clinical trial. Lasers Med Sci. May;33(4):719-727.

Nédélec, M.; McCall, A.; Carling, C.; Legall, F.; Berthoin, S. and Dupont, G. “Recovery in soccer: part II—recovery strategies,” Sports Medicine, vol. 43, no. 1, pp. 9–22, 2013.

Nédélec, M.; McCall, A.; Carling, C.; Legall, F.; Berthoin, S. and Dupont, G. Recovery in soccer: part I—post-match fatigue and time course of recovery,” Sports Medicine, vol. 42, no. 12, pp. 997–1015, 2012.

Notarnicola A, Covelli I, Maccagnano G, Marvulli R, Mastromauro L, Ianieri G, Boodhoo S, Turitto A, Petruzzella L, Farì G, Bianchi FP, Tafuri S, Moretti B. (2018) Extracorporeal shockwave therapy on muscle tissue: the effects on healthy athletes. J Biol Regul Homeost Agents. Jan-Feb;32(1):185-193.

Overmeyer, R.G., Driller, M.W. Pneumatic Compression Fails to Imporve Performance Recovery in Trained Cyclist. 2017. International Journal of Sports Physiology and Performance 13 (4): 1-21

Overmayer RG, Driller MW. A compressão pneumática não melhora a recuperação do desempenho em ciclistas treinados. Int J Sports Physiol Perform. 2018; 13 (4): 490–5.

Pinto HD, Vanin AA, Miranda EF, Tomazoni SS, Johnson DS, Albuquerque-Pontes GM, Aleixo IO Junior, Grandinetti VD, Casalechi HL, de Carvalho PT, Leal-Junior EC. (2016) Photobiomodulation Therapy Improves Performance and Accelerates Recovery of High-Level Rugby Players in Field Test: A Randomized, Crossover, Double-Blind, Placebo-Controlled Clinical Study. J Strength Cond Res. Dec;30(12):3329-3338.

Pinto HD, Casalechi HL, De Marchi T, et al. Photobiomodulation therapy combined with a static magnetic field applied in different moments enhances performance and accelerates muscle recovery in CrossFit® athletes: a randomized, triple-blind, placebo-controlled crossover trial. Oxid Med Cell Longev. 2022;2022: 9968428.

Poppendieck, W, Wegmann, M., Ferrauti, A., Kellmann, M., Pfeiffer, M. and Meyer, T. “Massage and performance recov- ery: a meta-analytical review,” Sports Medicine, vol. 46, no. 2, pp. 183–204, 2016.

Reid, M. B.; Haack, K. E.; Franchek, K. M.; Valberg, P. A.; Kobzik, L., and West, M. S. “Reactive oxygen in skeletal muscle. I. Intracellular oxidant kinetics and fatigue in vitro,” Journal of Applied Physiology, vol. 73, no. 5, pp. 1797–1804, 1992.

Reid, M. B. “Redox interventions to increase exercise perfor- mance,” The Journal of Physiology, vol. 594, no. 18, pp. 5125– 5133, 2016.

Sands WA, McNeal JR, Murray SR, Stone MH. (2015) Dynamic Compression Enhances Pressure-to-Pain Threshold in Elite Athlete Recovery: Exploratory Study. J Strength Cond Res. May;29(5):1263-72.

Schmitz C, Császár NB, Milz S, Schieker M, Maffulli N, Rompe JD, Furia JP. Efficacy and safety of extracorporeal shock wave therapy for orthopedic conditions: a systematic review on studies listed in the PEDro database. Br Med Bull. 2015;116(1):115-38. doi: 10.1093/bmb/ldv047. Epub 2015 Nov 18. PMID: 26585999; PMCID: PMC4674007.

Schwahn-Schreiber C, Breu FX, Rabe E, Buschmann I, Döller W, Lulay GR, Miller A, Valesky E, Reich-Schupke S. S1-Leitlinie Intermittierende Pneumatische Kompression (IPK, AIK) [S1 guideline on intermittent pneumatic compression (IPC)]. Hautarzt. 2018 Aug;69(8):662-673.

Smith, Michael M; Sommer, Allan J; Starkoff, Brooke E and Devor, Steven T. (2013) Cross Training-Based High Intensity Power Training Improves Maximal Aerobic Fitness and Body Composition.” Journal of Strength and Conditioning Research / National Strength & Conditioning Association 27 (11): 3159–72.

Speed, C. (2014) A systematic review of shockwave therapies in soft tissue conditions: focusing on the evidence. Br J Sports Med. 48(21):1538-42.

Sprey JWC, Ferreira T, de Lima MV, Duarte A, Jorge PB, Santili C. An epidemiological profile of Crossfit athletes in Brazil. Orthop J Sport Med. 2016;4:1–6.

Summitt RJ, Cotton RA, Kays AC, Slaven EJ. Shoulder injuries in individuals who participate in Crossfit training. Sports Health. 2016;8:541–6.

Tabata, I.; Nischimura, K.; Kouzaki, M.; Hirai, Y.; Ogita, F.; Miyachi, M. and Yamamoto, K. (1996) Effects of moderate-intensity endurance and high-intensity intermittent training on anaerobic capacity and VO2 max. Medicine & Science in Sports & Exercise 28(10), 1327-1330.

Tabata I, Irisawa K, Kouzaki M, et al. Meta-bolic profile of high intensity intermittentexercises.Med Sci Sports Exerc(1997);29:390–395.

Taheri P, Vahdatpour B, Andalib S. (2016) Comparative study of shock wave therapy and Laser therapy effect in elimination of symptoms among patients with myofascial pain syndrome in upper trapezius. Adv Biomed Res. Aug 30;5:138.

Tibana RA, de Almeida LM, Frade de Sousa NM, Nascimento DdC, Neto IVdS, de Almeida A, de Souza VC, Lopes MdFTPL, Nobrega OdT, Vieira DCL, Navalta JW and Prestes J (2016) Two Consecutive Days of Extreme Conditioning Program Training Affects Pro and Anti-inflammatory Cytokines and Osteoprotegerin without Impairments in Muscle Power. Front. Physiol. 7:260.

Tomazoni SS, Machado CDSM, De Marchi T, Casalechi HL, Bjordal JM, de Carvalho PTC, Leal-Junior ECP. Infrared Low-Level Laser Therapy (Photobiomodulation Therapy) before Intense Progressive Running Test of High-Level Soccer Players: Effects on Functional, Muscle Damage, Inflammatory, and Oxidative Stress Markers-A Randomized Controlled Trial. Oxid Med Cell Longev. 2019 Nov 16;2019:6239058. doi: 10.1155/2019/6239058. PMID: 31827687; PMCID: PMC6885272.

Vanin AA, Miranda EF, Machado, CSM, De Paiva PR, Albuquerque-Pontes GM, Casalechi HL, De Carvalho PTC, Leal-Junior ECP. (2016) What is the best moment to apply phototherapy when associated to a strength training program? A randomized, double- blinded, placebo-controlled trial. Lasers in Medical Science. 31, 1555-1564.

Vanin AA, Verhagen E, Barboza SD, et al. Photobiomodulation therapy for the improvement of muscular performance and reduction of muscular fatigue associated with exercise in healthy people: a systematic review and meta-analysis. Lasers Med Sci. 2018;33: 181-214.

Vollaard N. B.; Shearman J. P., and. Cooper, C. E. “Exercise- induced oxidative stress: myths, realities and physiological rel- evance,” Sports Medicine, vol. 35, no. 12, pp. 1045–1062, 2005.

Wang D, Wang Z, Zhang L, Li Z, Tian X, Fang J, Lu Q, Zhang X. (2018) Cellular ATP levels are affected by moderate and strong static magnetic fields. Bioelectromagnetics 39(5):352-360.

Weisenthal BM, Beck CA, Maloney MD, DeHaven KE, Giordano BD.(2014) Injury Rate and Patterns Among Crossfit Athletes. Orthop J Sports Med. Apr 25;2(4):2325967114531177.

Weston M, Siegler J, Bahnert A, et al. The application of differential ratings of perceived exertion to Australian Football League matches. J Sci Med Sport. 2015;18: 704-708.

Yamada M, Suzuki K, Kudo S, et. al. Raised plasma G-CSF and IL-6 after exercise may play a role in neutrophil mobilization into the circulation, J Appl Physiol 1985;92: 1789–1794, 2002; 10.1152/japplphysiol.00629.2001.

Zissler A, Steinbacher P, Zimmermann R, Pittner S, Stoiber W, Bathke AC, Sänger AM. Extracorporeal Shock Wave Therapy Accelerates Regeneration After Acute Skeletal Muscle Injury. Am J Sports Med. 2017 Mar;45(3):676-684. doi: 10.1177/0363546516668622. Epub 2016 Oct 13. PMID: 27729321.

Zuj KA, Prince CN, Hughson RL, Peterson SD. Superficial femoral artery blood flow with intermittent pneumatic compression of the lower leg applied during walking exercise and recovery. J Appl Physiol (1985). 2019 Aug 1;127(2):559-567.

https://games.Crossfit.com/history-of-the-games -acesso em 09/12/2021.

https://rallyfitness.com/blogs/news/the-business-of-Crossfit-an-update-on-new-market-research-2017 -acesso em 08/12/2021.

https://games.Crossfit.com/article/Crossfit-extends-contract-madison -acessado em 09/12/2021.

<http://www.ondesdechoc.eu/download.php?FNAME=1210983278_l894.upl&ANAME=BTL-shockwave_CAT_POR201.pdf>. - acesso em 09/06/2019

**APPENDIX I. Informed Consent Form**

**Informed Consent Form for Participation in Clinical Research**

Participant’s Name: __________________________________________

Address: __________________________________________

Contact Phone: __________________ City: ________________ ZIP Code: __________
E-mail: __________________________________________

**1. Title of the project:**

Project Title: *“Isolated and Combined Effects of Different Post-Exercise Recovery Strategies in Cross Training Athletes: A Randomized, Controlled, and Blinded Clinical Trial”*

**2. Objective:**

The aim of this project is to compare the effects of three different therapeutic resources—PBMT-sMF, IPC, and ESWT—on muscle recovery in Cross Training participants.

**3. Justification:**

The choice of the most effective therapeutic resource is of utmost importance for optimal results in the process of muscle fatigue recovery. Considering the high-intensity effort demanded in Cross Training practice, the use of resources that assist in the muscle recovery process is highly valuable, as it can ensure that participants are ready for subsequent training sessions (WODs) with reduced risk of injury.

Given the positive effects already demonstrated by some therapeutic resources, investigating whether these effects occur immediately after intense training or competition is necessary. Likewise, it is important to explore the best way to use each of these resources—whether in isolation or in combination—to optimize recovery. Thus, the advancement in the development and understanding of therapies or resources aimed at muscle recovery tailored to the needs of Cross Training participants is highly relevant for improved performance in this sport.

**4. Experimental Procedures:**

You are being invited to participate in this randomized, controlled, crossover, blinded clinical trial. Eligible participants will be male Cross Training practitioners, aged between 18 and 36 years, with at least one year of training experience, who will be randomized to different treatment orders (therapeutic resources) to be received each week.

You will take part in four weeks of research. Each week, you will undergo baseline (pre-WOD) assessments, followed by evaluations immediately post-WOD, as well as at 1h, 24h, and 48h after the exercise. Each assessment will last approximately 10 minutes and will include: A questionnaire to assess subjective perception of exertion (rating fatigue from 0 to 100 for legs and cardiorespiratory effort, ~1 minute); Blood sampling for subsequent analysis of muscle damage markers (lactate dehydrogenase – LDH) and oxidative stress/antioxidant activity markers (thiobarbituric acid reactive substances – TBARS, carbonylated proteins, catalase – CAT, and superoxide dismutase – SOD). Blood collection will be performed by a nurse (5 mL from the antecubital vein), lasting about 8 minutes; Functional evaluation, including two simple tests: (a) a free squat test (maximum repetitions in 1 minute) and (b) a countermovement jump test (maximum vertical jump).

You will then be asked to perform a WOD (workout of the day), consisting of three exercises: AirBike, Hang Squat Clean, and Box Jump Over (24 inches/60.69 cm). The sequence will follow the 21-15-9 scheme (21 reps in the first round, 15 in the second, and 9 in the last). The average duration will be about 15 minutes.

After the WOD, you will receive one of the following interventions (order randomized weekly): PBMT-sMF: Application of therapeutic light combined with static magnetic field, directly on the skin with light pressure, at eight predetermined sites on the thigh and leg muscles; ESWT: Application of ~6,000 acoustic pulses (micro-shocks) on thigh and leg muscles using a shockwave device; IPC: Use of pneumatic compression boots with four sequential chambers inflating along the legs and thighs; and Passive Recovery (PR): Resting in the supine position for 30 minutes. Each therapy will last ~30 minutes (PBMT-sMF: 32 min). Over the 4 weeks, you will receive all interventions.

**5. Risks or Expected Discomfort:**

You will be exposed to minimal risks. During all procedures, you will be supervised by a researcher. If you feel unwell, dizzy, or unstable, the evaluation will be immediately stopped. Blood collection may cause mild discomfort, local bruising, or pain similar to standard blood tests. Stored samples will be kept at −80 °C and properly discarded after analyses.

You may feel tired or experience temporary muscle discomfort during or after the WOD. As with any physical activity, there is a risk of muscle or joint injury.

**6. Protective Measures:**

In case of discomfort or injury during data collection, you will receive immediate assistance from the study nurse. If necessary, you will be referred to emergency care.

**7. Research Benefits:**

There will be no direct benefits for participants. However, the study results will provide relevant information about the most effective recovery strategies for reducing fatigue and improving performance in Cross Training athletes. All resources are non-invasive, non-pharmacological, and reported in the literature as safe.

**8. Alternative Methods:**

Not applicable.

**9. Withdrawal of Consent:**

Participation is voluntary, and you may withdraw at any time without penalty. For questions regarding risks, benefits, or procedures, you may contact the responsible researcher.

**10. Confidentiality:**

Only study-related data will be used, respecting confidentiality and ensuring that no participant will be personally identified.

**11. Expenses or Compensation:**

The study does not provide reimbursement or financial compensation. Travel expenses to the study site will be the responsibility of the participant.

**12. Study Site:**

The study will be conducted at Cross Training Sanja Box, Avenida Deputado Benedito Matarazzo, 8015 – Vila Bethania, São José dos Campos – SP. Phone: (12) 98126-2321.

**13. Research Ethics Committee (CEP):**

The study will be approved by the Ethics Committee of Universidade Nove de Julho – UNINOVE, which ensures compliance with Brazilian ethical standards (Resolutions CNS 466/2012 and 510/2016).

**Address: Rua Vergueiro, 235/249 – 12th floor – Liberdade – São Paulo – SP – CEP 01504-001.**

**Phone: (11) 3385-9010**

**E-mail: comitedeetica@uninove.br**

**Office hours: Monday to Friday – 11:30 am to 1:00 pm and 3:30 pm to 7:00 pm**

**14. Researchers’ Contact Information:**

**Principal Investigator:** Dr. Ernesto Cesar Pinto Leal Junior – Phone: (11) 99006-5829
**Responsible Student:** Paulo Henrique Gusmão – Phone: (11) 95268-4490

**15. Interim Events:**

Any adverse events arising during the study will be addressed appropriately.

São Paulo, ____ of _____________, 2020

**16. Consent Statement**

I, _______________________________________, after reading and understanding this informed consent form, acknowledge that my participation is voluntary and that I may withdraw at any time without prejudice. I confirm that I have received a copy of this form and authorize my participation in this research study, as well as the scientific use of data exclusively for this project.

**Signature of Participant:** ____________________________

(All pages must be initialed by the participant)

**17. Researcher’s Certification**

I, **Ernesto Cesar Pinto Leal Junior**, certify that:

a) This study will only begin after approval by the Research Ethics Committee (CEP).
b) The study respects human dignity and the protection of participants in accordance with ethical guidelines.
c) This study has scientific merit, and the research team is trained and qualified to perform the described procedures.

____________________________________
Ernesto Cesar Pinto Leal Junior

**ANEXO III - *Ratings of perceived exertion* (RPE)**

**
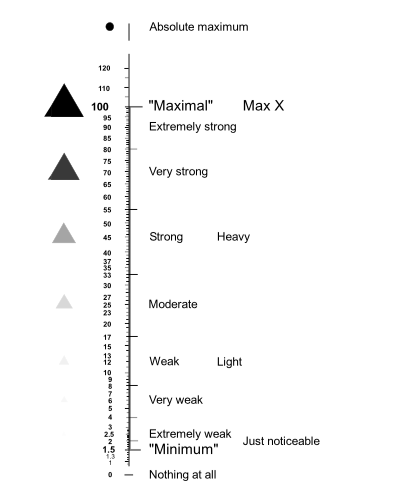
**


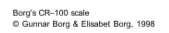

Supplement: S1 Dataset — This file contains all individual data points used in the analyses, including the data underlying all summary statistics presented in the manuscript. (DOCX) [file pone.0349880.s004.docx]
